# Differences in the characteristics and outcomes of STEMI versus NSTEMI cardiogenic shock: A systematic review and meta-analysis

**DOI:** 10.1097/MD.0000000000044951

**Published:** 2025-10-17

**Authors:** Zhichao Sheng, Binbin Chen

**Affiliations:** a Department of Cardiology, Xinchang County Traditional Chinese Medicine Hospital, Shaoxing City, Zhejiang Province, China.

**Keywords:** cardiogenic shock, clinical outcomes, meta-analysis, non ST-elevation myocardial infarction, ST-elevation myocardial infarction

## Abstract

**Background:**

There is limited evidence exploring the differences in characteristics and outcomes between patients with ST-segment elevation myocardial infarction (STEMI) and with non–ST-segment elevation myocardial infarction (NSTEMI) presenting with cardiogenic shock (CS).

**Methods:**

Medline, Google Scholar, and ScienceDirect databases were searched up to March 2025. Studies reporting data on STEMI-CS and NSTEMI-CS patient characteristics and clinical outcomes were included. Pooled risk ratios (odds ratios [ORs]) and standardized mean differences (SMDs) were calculated using random-effects models, and the *I*^2^ statistic measured heterogeneity. The risk of bias was assessed using the Newcastle–Ottawa Scale.

**Results:**

The pooled analysis of 12 studies demonstrated that the incidence of in-hospital mortality was comparable between STEMI-CS and NSTEMI-CS patients (pooled OR: 0.82, 95% confidence interval [CI]: 0.52–1.30, *I*^2^ = 98.7%, *P* < .001). However, STEMI-CS patients had a considerably lower age of presentation (pooled SMD: −0.54, 95% CI: −0.67 to −0.42, *I*^2^ = 96.9%, *P* < .001), as well as lower incidence of prior heart failure, prior myocardial infarction, and diabetes, compared to NSTEMI-CS patients (*P* < .05). Conversely, STEMI-CS was associated with shorter hospital stays (pooled SMD: −0.54, 95% CI: −0.77 to −0.31, *I*^2^ = 99.1%, *P* < .001).

**Conclusion:**

While the incidence of in-hospital mortality did not significantly differ between STEMI-CS and NSTEMI-CS patients, the study further emphasizes the importance of individualized treatment strategies based on myocardial infarction type and patient characteristics when managing CS patients.

## 1. Introduction

Acute myocardial infarction (AMI) has a prevalence of almost 3 million people worldwide and accounts for more than 1 million annual deaths in the United States alone.^[1]^ Severe myocardial dysfunction in AMI patients may result in cardiogenic shock (CS), which is characterized by inadequate tissue perfusion, leading to hemodynamic instability and organ failure.^[2,3]^ Despite advances in invasive and noninvasive cardiovascular techniques, CS remains one of the most life-threatening complications of AMI that occurs in around 5 to 10% of all patients with AMI and is associated with high (40–50%) mortality.^[4,5]^ Therefore, early identification and management of CS in AMI patients are crucial for improving their prognosis and survival.

Based on electrocardiography (ECG) findings, AMI is may be categorized as ST-elevation (STEMI) or non-ST-elevation (NSTEMI),^[6]^ which differ significantly in terms of pathophysiology and long-term outcomes.^[7,8]^ Existing literature has primarily focused on CS as a whole, without differentiating between the AMI subtypes, which limits our understanding of the distinct pathophysiological processes and their clinical implications.^[5]^

Recent studies that compared the clinical outcomes of myocardial infarction (MI) patients have shown that STEMI is linked to an increased in-hospital mortality, while NSTEMI is linked to poor long-term outcomes.^[9]^ While the incidence of CS is shown to be higher in STEMI patients, the clinical outcomes (in-hospital mortality, duration of hospital stay, and long-term prognosis) in the 2 groups are still unclear.^[10,11]^ While previous studies have identified several significant forecasters of in-hospital survival in AMI patients admitted with CS,^[12]^ there is still a need to further clarify the value of ECG patterns for prognostication.

Nursing professionals can substantially contribute to patient care and outcomes in managing AMI patients with CS. They participate in the evaluation, observation, and management of patients’ hemodynamic status, closely monitoring vital signs, cardiac rhythms, and therapeutic responses, administering medications like vasopressors and inotropic agents, and adjusting doses based on the patient’s response. Moreover, nurses collaborate with the multidisciplinary team to ensure a seamless continuum of care, helping patients and their families understand their disease, treatment options, and lifestyle changes by providing education and emotional support.^[13]^

This study aims to assess the differences in the characteristics and outcomes of STEMI and NSTEMI patients presenting with CS.

## 2. Methods

### 2.1. Research question

What is the difference in characteristics and outcomes in adults (>18 years) diagnosed with STEMI and NSTEMI-CS?

The latest PRISMA framework, published in 2020, was used for reporting.^[14]^ Ethical approval was not required since this review compiled the data that was available freely in selected databases.

### 2.2. Literature search

The literature search was conducted in Medline, ScienceDirect, and Google Scholar databases. English-language articles that were freely available from inception till March 2025 were included. Prospective and retrospective studies (cohort, case-control, and cross-sectional studies) of CS patients, as well as studies reporting baseline characteristics and relevant outcome parameters, were included. Additionally, studies reporting data on STEMI and NSTEMI patients were included. Conference abstracts, case reports, narrative reviews, randomized control trials, and editorials were excluded. All relevant characteristics and clinical outcomes reported in the included studies were extracted.

CS in all included studies was defined based on the previously recommended standard definition.^[15]^ Patients with CS were further divided into 2 categories, STEMI-CS and NSTEMI-CS, based on ECG findings.^[16]^

The latest universal definition of MI was used for the diagnosis of AMI.^[17,18]^ Any other electrocardiographic patterns in AMI patients were classified as NSTEMI.^[18]^

### 2.3. Collected data and outcomes of interest

The review included all baseline characteristics that were reported in common from the included studies such as age, female gender, heart rate, body mass index (BMI), Acute Physiology and Chronic Health evaluation score, sequential organ failure assessment score, left ventricular ejection fraction, troponin levels, comorbidities like current diabetes status, Charlson comorbidity index, prior MI, and previous heart failure.

The primary outcomes considered for the study were in-hospital mortality and mortality in the coronary care unit intensive care unit. The secondary outcomes included incidence of sepsis, respiratory failure, cardiac arrest, heart failure, invasive and noninvasive ventilator use, PCI use, length of CICU, and hospital stay in days.

### 2.4. Search strategy

Databases were screened using the medical subject heading terms: “CS” AND “baseline characteristics” AND “outcomes” OR “clinical outcomes” AND “STEMI” OR “NSTEMI” AND “observational studies” OR “retrospective studies” OR “prospective studies” along with free text terms as a filter (Supplementary File S1, Supplemental Digital Content, https://links.lww.com/MD/Q229).

### 2.5. Selection of studies

The primary investigators conducted the search, screened the titles and abstracts, and extracted the full text of relevant articles. The secondary investigators then extracted information on all baseline characteristics and clinical outcomes. The authors resolved any disagreement by discussion.

### 2.6. Data extraction

Two reviewers independently extracted study characteristics (authors, year, design, sample size, setting), patient demographics (age, sex, comorbidities), intervention details (STEMI‐CS vs NSTEMI‐CS definitions), and outcomes (in‐hospital mortality, PCI use) using a pilot‐tested, standardized data‐collection form. Discrepancies were resolved by discussion between the 2 reviewers. All extraction decisions including resolution of ambiguous or inconsistent entries were logged in a study‐specific audit trail. For studies reporting incomplete or unclear outcome data, we first contacted corresponding authors via e‐mail (up to 2 attempts) to request missing values.

### 2.7. Risk of bias assessment in included studies:

The 2 independent investigators assessed the risk of bias using the Newcastle–Ottawa scale (NOS) for observational studies. This scale comprises 7 items, classified into 3 domains: selection, comparability, and outcome, with the maximal score of 9.^[19]^

### 2.8. Statistical analysis and bias assessment

Microsoft Excel was used for data entry, and STATA 14.2 was used for analysis. Pooled differences were evaluated for continuous variables among STEMI and NSTEMI cases using the inverse variance method, which involved the standardized mean difference (SMD) and standard deviation. The differences in binary outcomes were summarized using odds ratios (OR) with 95% confidence intervals (CI) were calculated by the Mantel-Haenszel method. Finally, the pooled estimate was reported as SMD or OR with 95% CI based on the variable type. The *I*^2^ statistic and the Chi-square test of heterogeneity were used to assess the between-study variance. The heterogeneity was categorized as mild (*I*^2^ < 25%), moderate (*I*^2^ 25–75%), and substantial when *I*^2^ > 75%. To explore the source of heterogeneity, subgroup analysis was performed based on study region and meta-regression was performed based on potential covariates such as study region, study design, mean age, sample size, quality score and year of publication. A forest plot and funnel plot were used to graphically represent the pooled prevalence and publication bias. Egger test was done to test the asymmetry of funnel plot and *P*-value <.05 indicative of publication bias.

## 3. Results

### 3.1. Study selection

**Figure 1.**
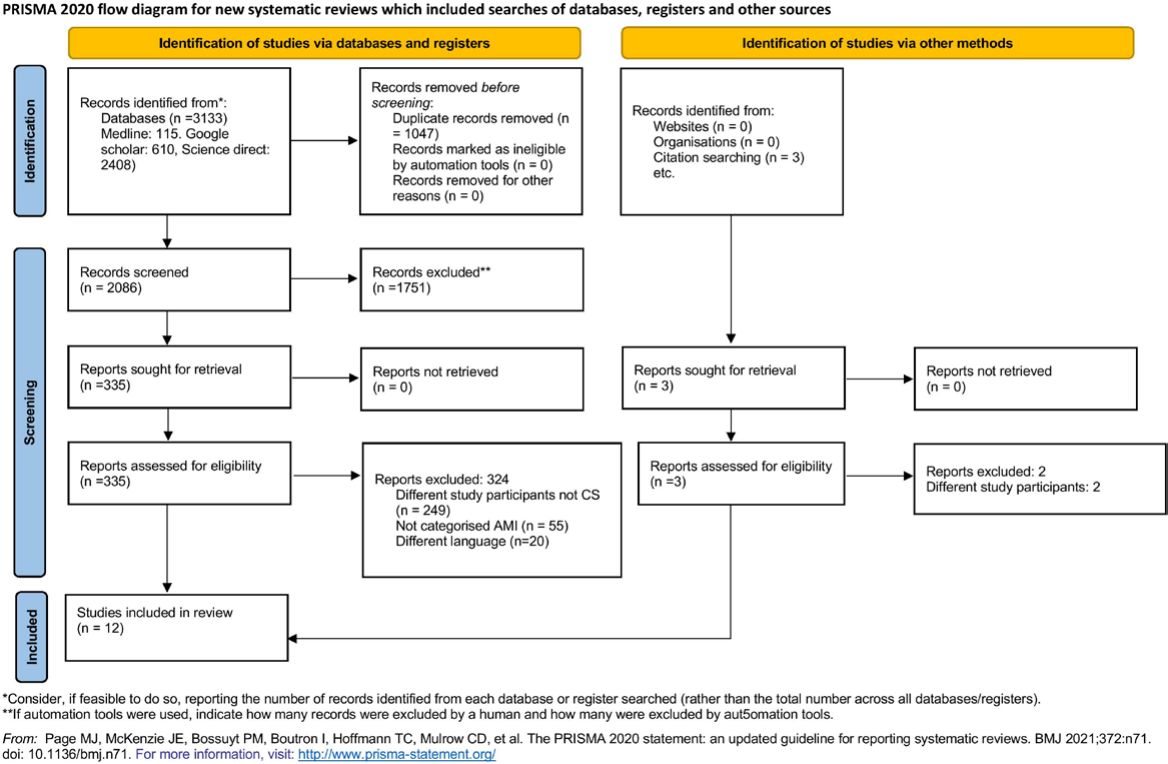
PRISMA 2020 flow diagram explaining the search flow. PRISMA = preferred reporting items for systematic reviews and meta-analyses.

Of 3133 identified articles, 1047 were removed as duplicates. An additional 1751 articles were eliminated after the title and abstract screening. Of the remaining 335 studies, 324 were excluded after the full-text evaluation. One study was obtained from a citation search. Finally, 12 articles were incorporated in the study ^[20–31]^. Figure 1 explains the PRISMA 2020 flow diagram.

### 3.2. Characteristics of the included studies

Table 1 summarizes the key characteristics of the 12 included studies. The studies were conducted across a diverse range of geographical settings, including the USA, Taiwan, Spain, various European countries, Egypt, South Korea, and Germany, with sample sizes ranging from 239 to over 15,000 patients. Most of the studies (10 out of 12) were retrospective, while a few were prospective or post hoc analyses of larger clinical trials or registries. The study settings varied, spanning single- and multicenter intensive care units, national databases, and specialized cardiac centers, with each study focusing on patients with AMI-CS in adults (typically ≥ 18 years).

**Table 1:** Characteristics of included studies, n = 12.

Study	Location	Sample size (NSTEMI/STEMI)	Study type	Study setting	Inclusion and exclusion criteria	Age (median and range/ mean (SD))	Independent variables	Outcomes	Quality of study (NOS)
Sarma et al 2023^26^	USA	581 (193/388)	Retrospective	Mayo Clinic St. Mary’s Hospital CICU database admitted between January 2007 and April 2018	Adult (age ≥ 18 yr) patients with an admission diagnosis of CS; postcardiotomy patients and patients with du able VADs or CMO. Patients with at available diagnoses and a TTE performed within 24-h of CICU admission were also included	Age: 69 (59, 77)	Demographic profiles, vital signs, admission laboratory data, vasoactive medicine use, mechanical support device use	CICU/hospital outcomes, organ failure assessment (SOFA) score, acute physiology and chronic health evaluation (APACHE)-III scores, Charlson comorbidity index (CCI) to quantify the severity of illness, ECHO parameters, and mortality	6
Tsai et al 2022^31^	Taiwan	1175 (460/715)	Retrospective	Chang Gung research database (CGRD), of the Chang Gung memorial hospital health system between 2000–2018	Patients admitted in emergency department with AMI complicated by cardiogenic shock events requiring inotropic agent, intra-aortic balloon pump (IABP), or extracorporeal membrane oxygenation (ECMO) in the first 3 d were enrolled in the study.	67.65 ± 13.86	Demographic profiles, vital signs, treatment provided, comorbidity, vasoactive medicine use	The primary outcomes were 30-d mortality or mortality at index hospitalization. Secondary outcomes included new-onset hemodialysis, new-onset stroke, major bleeding, gastrointestinal (GI) bleeding, pneumonia, or sepsis within 30 d after the index admission or during the index hospitalization. 1-yr follow up outcomes such as recurrent myocardial infarction, coronary revascularization, death, stroke, or bleeding events.	7
Martinez et al 2022^30^	Spain	239 (49/190)	Retrospective	Patients admitted in intensive cardiac care unit (ICCU) of a single tertiary university hospital in Barcelona (Spain)	AMI-CS who were ad- mitted to the intensive cardiac care unit (ICCU)	69.7 (11.6)	Baseline characteristics, clinical presentation, laboratory values, coronary anatomy, revascularization features, procedures and medical therapies, and in-hospital complications	Follow-up was performed at 3 specified time points (1, 3, and 5 yr after admission) by telephone contact and electronic patient record review to get information on vital status, cause of death, and need for readmission due to cardiovascular cause	6
Pahuja et al 2022^21^	Europe	1110 (731/379)	Retrospective	Data obtained from 17 sites from the cardiogenic shock working group (CSWG) registry	AMI-CS who were ad- mitted to the intensive cardiac care unit (ICCU)	NA	Baseline characteristics, clinical characteristics, hemodynamic, and metabolic profiles	Mortality, cardiac arrest, and SCAI score	5
Zeitouni et al 2020^28^	European countries	567 (195/372)	Retrospective	post hoc analysis included all the patients of the CULPRIT-SHOCK (culprit lesion only PCI versus multivessel PCI in cardiogenic shock) trial for whom the ECG presentation was collected	AMI-CS who were ad- mitted to the intensive cardiac care unit (ICCU)	70.0 [60.0–78.0]	Baseline characteristics, clinical presentation, laboratory values, BMI, comorbidity, cardiac and ECHO parameters	30-d and 1-yr all-cause death of patients admitted with cardiogenic shock related to NSTE-MI and STEMI-MI.	6
Liakopoulos et al 2019^29^	Germany	430 (227 + 243)	Retrospective	The surgical myocardial infarction registry analyzed by 4 university-affiliated cardiac surgery centers within North-Rhine-Westphalia	Adult patients (age > 18 yr) requiring CABG for ACS	NA	Baseline characteristics, clinical presentation, laboratory values, operative characteristics	All-cause mortality was the primary outcomes measure. MACE outcomes, clinical outcomes, need of respiratory support and length of hospital stay	5
Anderson et al 2015^27^	USA	17,536 (11,406/6130)	Retrospective	The national cardiovascular data registry’s, acute coronary treatment and intervention outcomes network registry–get with the guidelines (ACTION registry–GWTG) encompassing data from > 600 hospitals in USA	Adult patients (age > 18 yr) with diagnosed CD	NA	Baseline demographics, clinical characteristics, and outcomes were displayed by MI type or by the presence or absence of shock. These variables were further stratified by the timing of shock for each MI subtype.	Mortality, clinical outcomes, and treatment outcomes	7
Diaz-Arocutipa et al 2025^23^	USA	150,395 (62,915/87,480)	Retrospective	National Inpatient Sample (NIS) database (2016–2019)	Patients hospitalized with a primary diagnosis of acute myocardial infarction complicated by cardiogenic shock.	68 (60–77)	Demographics, comorbidities (e.g., hypertension, diabetes, atrial fibrillation, previous MI), hospital characteristics, treatment modalities (PCI, CABG, mechanical circulatory support, mechanical ventilation, renal replacement therapy).	Primary outcome: in-hospital mortality. Secondary outcomes: major bleeding, acute stroke, mechanical complications of MI (ventricular rupture, papillary muscle rupture), length of hospital stay.	6
Taha et al 2024^22^	Egypt	529 (78/142)	Prospective	Egyptian multicenter registry (6 tertiary referral centers)	Patients aged ≥18 yr with cardiogenic shock (CS) fulfilling clinical criteria: SBP < 90 mm Hg for > 30 min, need for vasopressors, cardiac index < 2.2 L/min/m^2^, or evidence of organ hypoperfusion.	62 ± 14.2	Demographic characteristics, comorbidities (diabetes, hypertension, renal impairment), laboratory parameters (creatinine, leucocyte count, ALT), inotropic/vasopressor use (norepinephrine, epinephrine, dopamine), coronary interventions (PCI, CABG), and STEMI location (anterior vs inferior).	Primary outcome: 30-d all-cause mortality. Secondary outcomes: predictors of mortality (renal function decline, resuscitated cardiac arrest, use of multiple inotropes/vasopressors), and impact of STEMI location on survival.	5
Jeon et al 2023^20^	South Korea	184 (70/114)	Retrospective	Multicenter registry (12 tertiary centers in South Korea)	Included female patients aged > 19 yr who underwent PCI for AMI complicated by cardiogenic shock.	72.89 ± 11.60 yr (STEMI), 75.81 ± 8.69 yr (NSTEMI)	Demographics, comorbidities (hypertension, diabetes, dyslipidemia), prior cardiovascular history (previous MI, PCI), hemodynamic parameters, laboratory markers (troponin, creatinine, NT-proBNP), coronary anatomy (culprit lesion, number of vessels affected), and treatment variables (PCI characteristics, thrombus aspiration, mechanical circulatory support use).	Primary outcome: Major adverse cardiac events (MACE) at 12 mo (composite of cardiac death, MI, or repeat revascularization). Secondary outcomes: In-hospital mortality, cardiac mortality, all-cause mortality, and rehospitalization due to heart failure.	7
Sinha et al 2023^24^	USA	1110 (379/731)	Retrospective	Cardiogenic Shock Working Group (CSWG) registry (17 centers in the USA)	Adult patients admitted with cardiogenic shock (CS) complicating acute myocardial infarction (AMI).	65.6 ± 12.5	Demographics, comorbidities (hypertension, diabetes, chronic kidney disease), hemodynamic parameters (SBP, cardiac index), metabolic markers (serum creatinine, lactate, ALT), treatment intensity (vasopressors, inotropes, mechanical circulatory support), and clinical trajectory (SCAI shock stage progression).	Primary outcome: in-hospital mortality. Secondary outcomes: impact of cardiac arrest (higher mortality in NSTEMI-CS vs STEMI-CS), drug and device utilization trends during hospitalization.	6
Schupp et al 2024^25^	Germany	273 (32/102)	Prospective	Single-center registry (University Medical Center Mannheim, Germany)	Consecutive patients presenting with cardiogenic shock (CS) from 2019–2021. Diagnosis of CS was based on ESC criteria (SBP < 90 mm Hg for > 30 min or need for vasopressor/inotropic therapy, with signs of end-organ hypoperfusion).	72 (65–81)	Demographics, comorbidities (hypertension, diabetes, atrial fibrillation, chronic kidney disease), hemodynamic parameters (SBP, heart rate, lactate, creatinine), coronary angiography findings (need for mechanical circulatory support Impella), and resuscitation history	Primary outcome: 30-d all-cause mortality. Secondary outcomes: impact of STEMI vs NSTEMI on mortality	7

ALT = alanine aminotransferase, APACHE = acute physiology and chronic health evaluation, CCI = Charlson comorbidity index, CICU = cardiac intensive care unit, MACE = major adverse cardiac events, MI = myocardial infarction, NSTEMI = non-ST-elevation myocardial infarction, PCI = percutaneous coronary intervention, SBP = systolic blood pressure, SCAI = society for cardiovascular angiography and interventions, STEMI = ST-elevation myocardial infarction.

Independent variables examined across studies included demographic profiles, clinical and laboratory parameters, hemodynamic and metabolic profiles, echocardiographic findings, and other relevant factors. Outcomes of interest were primarily centered on mortality metrics (in-hospital, 30-day, or long-term mortality), with several studies also reporting additional adverse events such as major bleeding, stroke, and rehospitalization rates.

As shown in Table 2, 9 of the 12 included studies had high quality (NOS score ≥ 6).

**Table 2:** Risk of bias assessment amongst included studies, n = 12.

Study	Selection	Comparability	Outcome	Quality of study (NOS)
Sarma et al, 2023^26^	2 points	2 points	2 points	6
Tsai et al, 2022^31^	3 points	2 points	2 points	7
Martinez et al, 2022^30^	2 points	2 points	2 points	6
Pahuja et al, 2022^21^	2 points	1 point	2 points	5
Zeitouni et al, 2020^28^	2 points	2 points	2 points	6
Liakopoulos et al, 2019^29^	1 point	2 points	2 points	5
Anderson et al, 2015^27^	3 points	2 points	2 points	7
Diaz-Arocutipa et al, 2025^23^	2 points	2 points	2 points	6
Taha et al, 2024^22^	2 points	1 point	2 points	5
Jeon et al, 2023^20^	3 points	2 points	2 points	7
Sinha et al, 2023^24^	2 points	2 points	2 points	6
Schupp et al, 2024^25^	3 points	2 points	2 points	7

NOS = Newcastle–Ottawa scale.

### 3.3. Difference in outcome parameters between STEMI-CS and NSTEMI-CS patients

**Figure 2.**
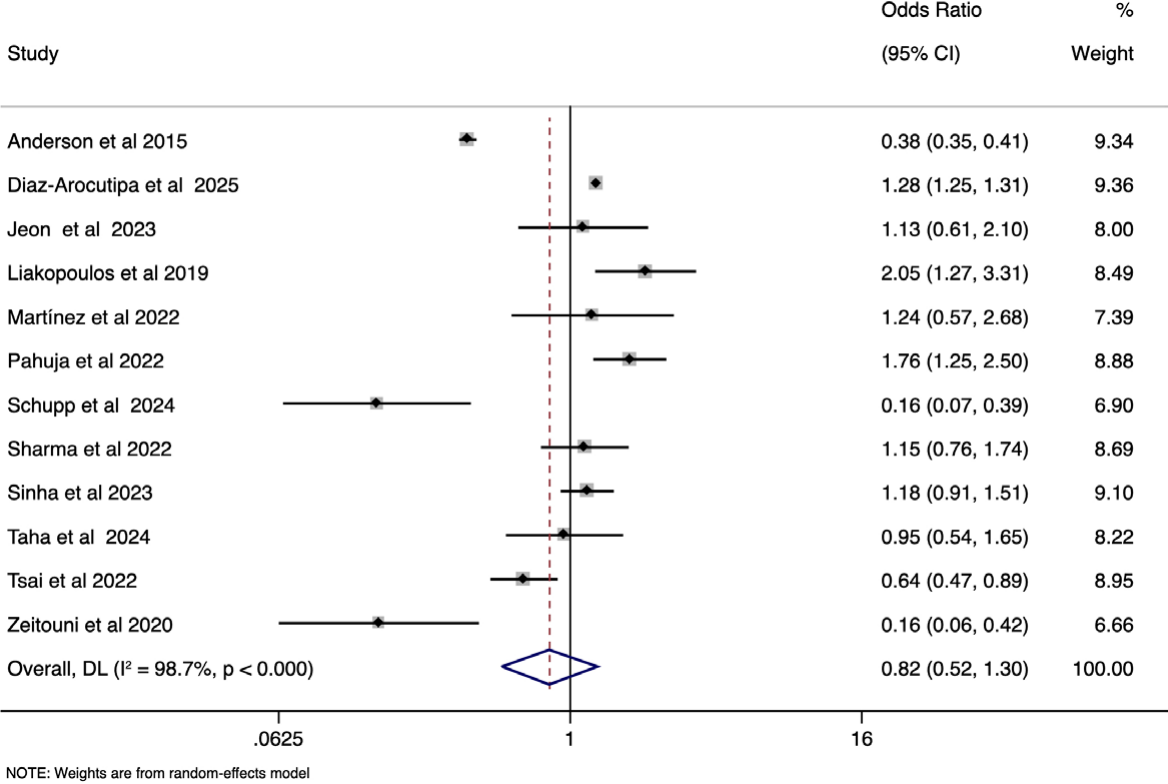
Forest plot showing the difference in incidence of mortality between STEMI-CS and NSTEMI-CS. CS = cardiogenic shock, STEMI = ST-elevation, NSTEMI = non-ST-elevation.

**Figure 3.**
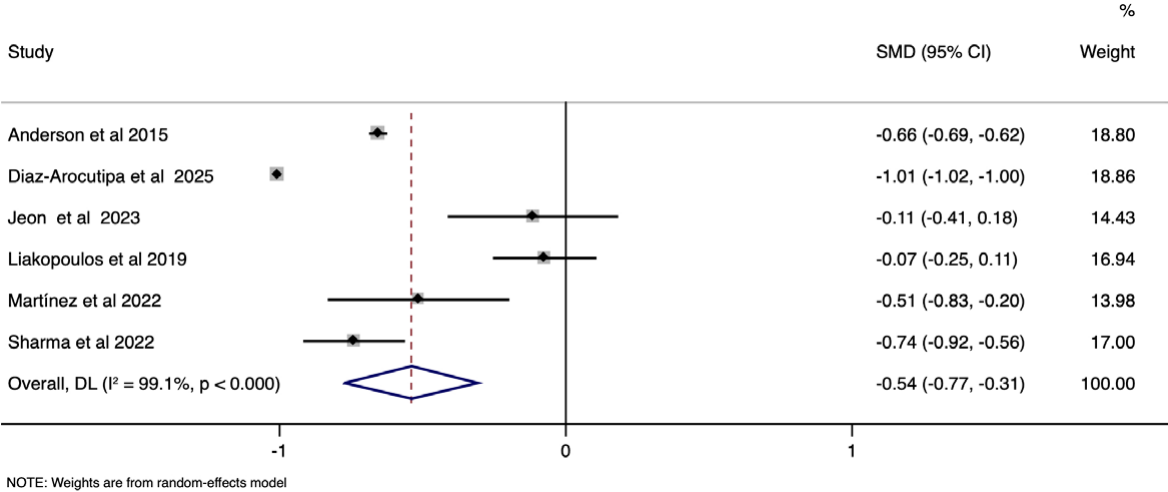
Forest plot showing the difference in hospital stay between STEMI-CS and NSTEMI-CS. CS = cardiogenic shock, STEMI = ST-elevation, NSTEMI = non-ST-elevation.

STEMI-CS and NSTEMI-CS patients did not vary much in the rates of in-hospital mortality (pooled OR 0.82, 95% CI: 0.52–1.30), (*I*^2^ = 98.7%, *P* < .001) [Fig. 2] need for PCI (pooled OR 1.97, 95% CI: 0.46–8.42), (*I*^2^ = 99.9%, *P* < .001) [Supplementary file S2, Supplemental Digital Content, https://links.lww.com/MD/Q230], need for invasive ventilator (pooled OR 1.00, 95% CI: 0.70–1.45), (*I*^2^ = 84.6%, *P* < .001) [Supplementary file S3, Supplemental Digital Content, https://links.lww.com/MD/Q230], and CICU stay duration in days (SMD of −0.22; 95% CI: −0.48–0.04), (*I*^2^ = 86.5%, *P* < .001) [Supplementary file S4, Supplemental Digital Content, https://links.lww.com/MD/Q230]. However, STEMI-CS patients had a significantly lower incidence of sepsis (pooled OR 0.40, 95% CI: 0.29–0.57), (*I*^2^ = 42.8%, *P* .19) [Supplementary file S5, Supplemental Digital Content, https://links.lww.com/MD/Q230], and required shorter hospital stay (SMD of −0.54; 95% CI: −0.77 to −0.31), *I*^2^ = 99.1%, *P* < .001) [Fig. 3] than NSTEMI-CS. There was a significant variability in the rates of cardiac arrest (pooled OR 1.78, 95% CI: 0.98–3.24), (*I*^2^ = 84.3%, *P* < .001) [Supplementary file 6, Supplemental Digital Content, https://links.lww.com/MD/Q230].

### 3.4. Difference in baseline characteristics between STEMI-CS and NSTEMI-CS patients

**Figure 4.**
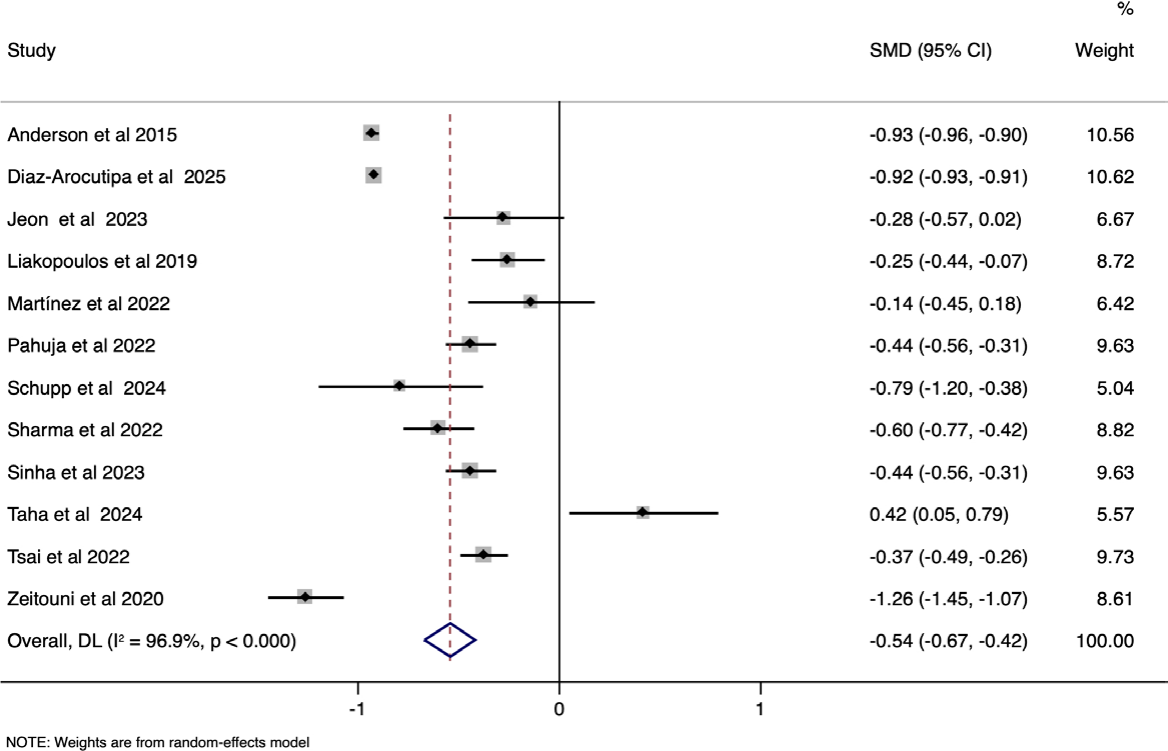
Forest plot showing the difference in age of mortality between STEMI-CS and NSTEMI-CS. CS = cardiogenic shock, STEMI = ST-elevation, NSTEMI = non-ST-elevation.

**Figure 5.**
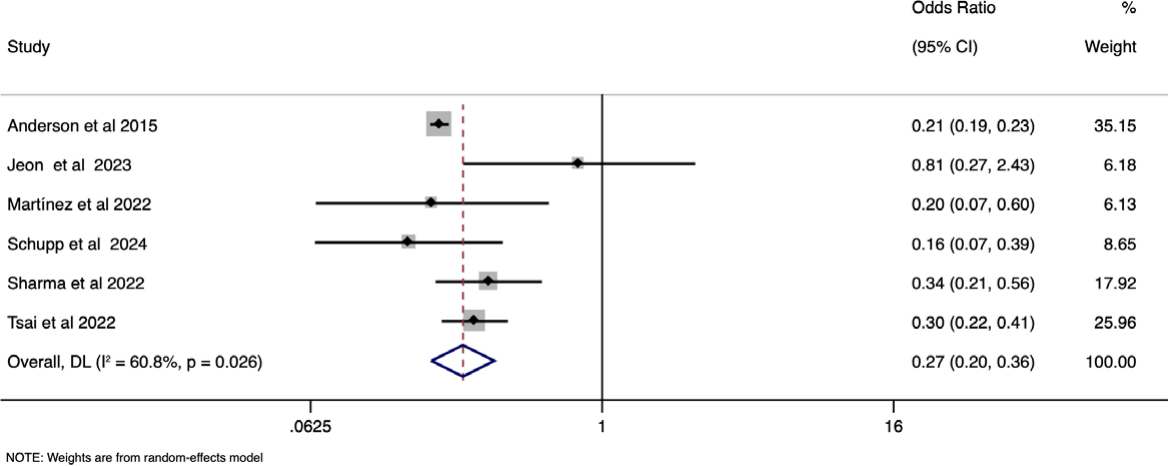
Forest plot showing the difference in prior heart failure between STEMI-CS and NSTEMI-CS. CS = cardiogenic shock, STEMI = ST-elevation, NSTEMI = non-ST-elevation.

**Figure 6.**
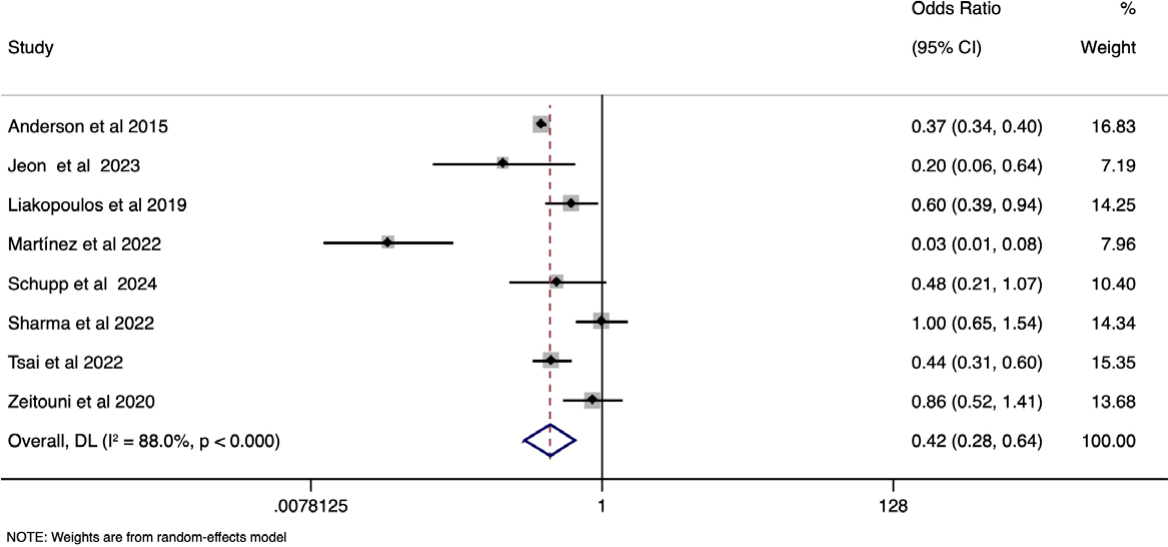
Forest plot showing the difference in prior MI between STEMI-CS and NSTEMI-CS. CS = cardiogenic shock, STEMI = ST-elevation, NSTEMI = non-ST-elevation.

STEMI-CS patients had a significantly lower age of presentation when compared to NSTEMI-CS cases, with the pooled SMD of −0.54 (95% CI: −0.67 to −0.42), (*I*^2^ = 96.9%, *P* < .001) [Fig. 4]. Female gender distribution (pooled OR 0.96, 95% CI: 0.80–1.16), (*I*^2^ = 89.4%, *P* < .001) [Supplementary file S7, Supplemental Digital Content, https://links.lww.com/MD/Q230], left ventricular ejection fraction distribution (SMD of 0.02; 95% CI: −0.27–0.31), (*I*^2^ = 95.7%, *P* < .001) [Supplementary file S8, Supplemental Digital Content, https://links.lww.com/MD/Q230], heart rate (SMD of −0.23; 95% CI: −0.66–0.19), (*I*^2^ = 98.2%, *P* < .001) [Supplementary file S9, Supplemental Digital Content, https://links.lww.com/MD/Q230] were comparable between the STEMI-CS and STEMI-CS. However, the STEMI-CS group reported lesser incidence of prior heart failure (pooled OR 0.27, 95% CI: 0.20–0.36), (*I*^2^ = 60.8%, *P* .03), [Fig. 5] prior MI (pooled OR 0.42, 95% CI: 0.28–0.64), (*I*^2^ = 88%, *P* < .001) [Fig. 6], diabetes (, (pooled OR 0.36, 95% CI: 0.25–0.52), (*I*^2^ = 37.9%, *P* .15) [Supplementary file S10, Supplemental Digital Content, https://links.lww.com/MD/Q230], and lower BMI (WMD of −0.44; 95% CI: −0.80 to −0.07) , (*I*^2^ = 16.1%, *P* .30) [Supplementary file S11, Supplemental Digital Content, https://links.lww.com/MD/Q230], when compared to the NSTEMI-CS patients.

### 3.5. Publication bias

Publication bias assessment was done for in-hospital mortality outcomes. The funnel plot (Supplementary file S12, Supplemental Digital Content, https://links.lww.com/MD/Q230) showed symmetry among the included studies, as confirmed by Egger test (*P* = .31), indicating the absence of publication bias.

### 3.6. Subgroup analysis and meta-regression

#### 3.6.1. In-hospital mortality

In subgroup analyses by WHO region, the pooled OR for in-hospital mortality in STEMI-CS versus NSTEMI-CS was 0.894 (95% CI: 0.402–1.989; *I*^2^ = 99.6%) in America, 0.815 (95% CI: 0.574–1.157; *I*^2^ = 37.8%) in Asia, and 0.699 (95% CI: 0.277–1.768; *I*^2^ = 91.3%) in Europe. The test for between-region differences was non-significant (*Q* = 0.16, df = 2, *P* = .925), indicating no evidence of effect modification by region (Supplementary file S13, Supplemental Digital Content, https://links.lww.com/MD/Q230). Region did not significantly moderate mortality (Adj R^2^ = –30.8%; joint *P* = .925), indicating no explained heterogeneity. Study design was non-significant (Adj R^2^ = 4.4%; *P* = .248). Quality score was the only significant predictor (Adj R^2^ = 44.5%; *P* = .045), explaining nearly half the residual variance. Mean age had no effect (Adj R^2^ = –5.1%; *P* = .380), nor did sample size (Adj R^2^ = –12.0%; *P* = .643). Year of publication together explained 46.1% of between-study variance but was not significantly associated with effect size (joint *P* = .209).

#### 3.6.2. Need for PCI

In region-specific analyses of PCI use, the pooled OR for STEMI-CS versus NSTEMI-CS was 2.157 (95% CI: 0.260–17.883; *I*^2^ = 99.9%) in America, 1.665 (95% CI: 0.728–3.811; *I*^2^ = 74.5%) in Asia, and 1.784 (95% CI: 0.173–18.441; *I*^2^ = 94.2%) in Europe. The test for between-region differences was non-significant (*Q* = 0.05, df = 2, *P* = .975), indicating no evidence of effect modification by region (Supplementary file S14, Supplemental Digital Content, https://links.lww.com/MD/Q230). Meta-regression did not show significant association with any variables or explain the heterogeneity based on any of the covariates (study region, sample size, mean age, year of publication, quality score and study design).

Subgroup analysis and meta-regression could not be performed for need of invasive ventilation and CICU duration due to limited number of studies.

#### 3.6.3. Sensitivity analysis

Sensitivity analysis (Supplementary files S15–S18, Supplemental Digital Content, https://links.lww.com/MD/Q230) showed that there was no significant variation in the pooled effect size due to single study effects, indicating the studies are robust to outliers.

## 4. Discussion

The study showed similar incidences of in-hospital mortality in STEMI-CS and NSTEMI-CS patients. However, STEMI-CS was associated with significantly lower rates of sepsis and cardiac arrest and shorter length of hospital stay. Additionally, there was a considerable difference in baseline characteristics such as age, prior history of heart failure and MI, BMI, and diabetes disease status between the STEMI-CS and NSTEMI-CS groups.

Studies showed that NSTEMI patients present with more cardiovascular risk factors,^[10,20]^ probably due to extensive coronary involvement. However, such patterns have not yet been reported in AMI-CS patients. Furthermore, this is the first review to explore different clinical characteristics and outcomes in STEMI and NSTEMI patients with complicating CS, utilizing the highest form of evidence.

In-hospital mortality was comparable in the 2 types of patients in this study. This result confirms the finding by Pahuja et al,^[21]^ which also reported comparable mortality rates across both study groups. However, it is important to note the significant heterogeneity observed across the studies (*I*^2^ = 89.0%), indicating substantial variability. The lack of consensus regarding the impact of MI type on mortality in CS patients warrants further investigation and may be attributed to variations in patient characteristics, treatment strategies, and follow-up protocols across different healthcare settings.

This study demonstrated that NSTEMI-CS patients presented at a younger age than STEMI-CS patients. This finding suggests that NSTEMI-CS primarily affects older individuals and is consistent with earlier studies.^[4,22]^ As advancing age is frequently associated with higher comorbidities and fragility, the greater age of NSTEMI-CS patients may influence treatment choices and resource allocation. Furthermore, this review demonstrated that, compared to the NSTEMI-CS group, STEMI-CS patients had a significantly lower incidence of prior heart failure, MI, diabetes, and a lower BMI. These findings suggest that STEMI-CS patients could have a relatively healthier baseline cardiovascular profile than NSTEMI cases. On the contrary, compared to STEMI-CS patients, NSTEMI-CS was linked to a higher incidence of sepsis and longer hospital admissions. This finding raises the possibility of a link between NSTEMI-CS and an increased systemic inflammatory response in the body, which could lead to worse outcomes and a longer recovery period.

The results indicate a low likelihood that selective reporting or small‐study effects materially influenced our summary estimates for secondary interventions. Nevertheless, we acknowledge that the relatively small number of studies available for certain endpoints may reduce the power of bias‐detection methods. Taken together, the absence of detectable publication bias across both primary and secondary outcomes strengthens the credibility and robustness of our meta‐analytic findings.

## 5. Clinical implications

Beyond statistical comparisons, these findings carry important clinical implications for the management of CS complicating STEMI versus NSTEMI. First, although the overall odds of in‐hospital mortality did not differ significantly between STEMI‐CS and NSTEMI‐CS (OR 0.82, 95% CI: 0.52–1.30), the high absolute mortality rates observed in both groups (ranging from 20–50% in contemporary cohorts) underscore the need for rapid identification and escalation of care in all patients with MI complicated by shock. Clinicians should therefore maintain a low threshold for early invasive monitoring (e.g., pulmonary artery catheterization) and initiation of vasopressor or inotropic support as soon as shock is recognized, regardless of infarct type.

Second, our subgroup analyses suggested considerable regional variability in PCI use – though differences were not statistically significant, practice patterns ranged from predominantly emergent angiography in Europe to more variable approaches in Asia and the Americas. From a clinical standpoint, this highlights the value of standardized “shock team” protocols, in which interventional cardiology, critical care, and cardiac surgery collaborate to ensure that mechanical circulatory support (e.g., intra‐aortic balloon pump, Impella, or extracorporeal membrane oxygenation) is deployed alongside revascularization when indicated. Such multidisciplinary models have been associated with improved survival in observational cohorts and are now incorporated into international guidelines for the management of STEMI complicated by shock.

Third, the strong inverse association between study quality and effect size in our meta‐regression (quality score β = –0.58, *P* = .045) serves as a reminder that real‐world clinical outcomes may be even more nuanced than those captured in retrospective registry analyses. High‐quality randomized data will be needed to clarify whether differential infarct‐related management strategies such as complete versus culprit‐only PCI, timing of revascularization, or use of advanced support devices translate into meaningful survival benefits. In the interim, clinicians should individualize revascularization decisions based on hemodynamic stability, infarct complexity, and comorbidity burden rather than relying solely on presentation subtype.

## 6. Strengths and limitations

This review is the first to evaluate different baseline characteristics and outcomes of STEMI-CS and NSTEMI-CS. The review included 12 studies with a substantial sample size. Despite these strengths, the study has several limitations, including the high interstudy heterogeneity, which suggests potential variability in patient populations, interventions, and outcomes. Further studies across various healthcare settings should address the variability in treatment strategies, patient characteristics, and follow-up protocols.

## 7. Conclusions and Recommendations

This study provides insight into the characteristics and outcomes of CS patients with different ECG parameters. While it reported similar in-hospital mortality in the 2 groups, substantial differences were observed in age, comorbidities, and clinical outcomes. These results further emphasize the importance of considering MI type while managing CS. Based on these findings, clinicians should carefully consider each patient’s unique characteristics, such as age, past cardiovascular history, and comorbidities, when deciding the management of AMI-CS patients.

## Author contributions

**Conceptualization:** Zhichao Sheng.

**Data curation:** Zhichao Sheng, Binbin Chen.

**Formal analysis:** Zhichao Sheng, Binbin Chen.

**Methodology:** Zhichao Sheng, Binbin Chen.

**Writing – original draft:** Zhichao Sheng.

**Writing – review & editing:** Zhichao Sheng.

## Supplementary Material




