# Supplementary material for: Differences in the characteristics and outcomes of STEMI versus NSTEMI cardiogenic shock: A systematic review and meta-analysis
Source: Medicine (Baltimore). 2025 Oct 17;104(42):e44951. doi: 10.1097/MD.0000000000044951 (PMC12537256; doi:10.1097/MD.0000000000044951)
Supplement: Supplementary file 1 [file medi-104-e44951-s001.docx]

**Differences in the characteristics and outcomes of STEMI vs NSTEMI cardiogenic shock: A systematic review and meta-analysis**

Zhichao Sheng, MD, Binbin Chen^*^, MD

Department of Cardiology, Xinchang County Traditional Chinese Medicine Hospital, Shaoxing City, Zhejiang Province, China

^*^Corresponding author: Binbin Chen, Department of Cardiology, Xinchang County Traditional Chinese Medicine Hospital, No. 188, Nineteen Peaks Road, Xinchang County, Shaoxing City, Zhejiang Province, China

Email: [ted1192@126.com](mailto:ted1192@126.com)

**ABSTRACT**

There is limited evidence exploring the differences in characteristics and outcomes between patients with ST-segment elevation myocardial infarction (STEMI) and with non-ST-segment elevation myocardial infarction (NSTEMI) presenting with cardiogenic shock (CS). Medline, Google Scholar, and ScienceDirect databases were searched up to March 2025. Studies reporting data on STEMI-CS and NSTEMI-CS patient characteristics and clinical outcomes were included. Pooled risk ratios (ORs) and standardized mean differences (SMDs) were calculated using random-effects models, and the I2 statistic measured heterogeneity. The risk of bias was assessed using the Newcastle-Ottawa Scale (NOS). The pooled analysis of 12 studies demonstrated that the incidence of in-hospital mortality was comparable between STEMI-CS and NSTEMI-CS patients (pooled OR 0.82, 95% CI: 0.52 to 1.30, I2 = 98.7%, p < 0.001). However, STEMI-CS patients had a considerably lower age of presentation (pooled SMD -0.54, 95% CI: -0.67 to -0.42, I^2^ = 96.9%, p < 0.001), as well as lower incidence of prior heart failure, prior MI, and diabetes, compared to NSTEMI-CS patients (p <0.05). Conversely, STEMI-CS was associated with shorter hospital stays (pooled SMD -0.54, 95% CI -0.77 to -0.31, I^2^ = 99.1%, p < 0.001). While the incidence of in-hospital mortality did not significantly differ between STEMI-CS and NSTEMI-CS patients, the study further emphasizes the importance of individualized treatment strategies based on MI type and patient characteristics when managing CS patients.

**Keywords:** Non ST elevation myocardial infarction, Clinical outcomes, Meta-analysis, Cardiogenic shock, ST elevation myocardial infarction;

**Prospero**: CRD420251005920

**ABBREVIATIONS**

Acute myocardial infarction (AMI)

Cardiogenic shock (CS)

Electrocardiography (ECG)

ST-elevation (STEMI)

Non-ST-elevation (NSTEMI)

Myocardial infarction (MI)

Preferred Reporting Items for Systematic Reviews and Meta-Analyses (PRISMA)

Body mass index (BMI)

Acute Physiology and Chronic Health Evaluation (APACHE)

Sequential Organ Failure Assessment (SOFA)

Left ventricular ejection fraction (LVEF)

Charlton comorbidity index (CCI)

Coronary care unit intensive care unit (CCUICU)

Medical subject heading (MeSH)

Newcastle-Ottawa Scale (NOS)

Standardized mean difference (SMD)

Odds ratios (OR)

Confidence intervals (CI)

**INTRODUCTION**

Acute myocardial infarction (AMI) has a prevalence of almost 3 million people worldwide and accounts for more than 1 million annual deaths in the United States alone ^1^. Severe myocardial dysfunction in AMI patients may result in cardiogenic shock (CS), which is characterised by inadequate tissue perfusion, leading to hemodynamic instability and organ failure ^2,3^. Despite advances in invasive and non-invasive cardiovascular techniques, CS remains one of the most life-threatening complications of AMI that occurs in around 5-10% of all patients with AMI and is associated with high (40-50%) mortality ^4,5^. Therefore, early identification and management of CS in AMI patients are crucial for improving their prognosis and survival.

Based on electrocardiography (ECG) findings, AMI is may be categorized as ST-elevation (STEMI) or non-ST-elevation (NSTEMI) ^6^, which differ significantly in terms of pathophysiology and long-term outcomes ^7,8^. Existing literature has primarily focused on CS as a whole, without differentiating between the AMI subtypes, which limits our understanding of the distinct pathophysiological processes and their clinical implications ^5^.

Recent studies that compared the clinical outcomes of myocardial infarction (MI) patients have shown that STEMI is linked to an increased in-hospital mortality, while NSTEMI is linked to poor long-term outcomes ^9^. While the incidence of CS is shown to be higher in STEMI patients, the clinical outcomes (in-hospital mortality, duration of hospital stay, and long-term prognosis) in the two groups are still unclear. ^10,11^. While previous studies have identified several significant forecasters of in-hospital survival in AMI patients admitted with CS ^12^, there is still a need to further clarify the value of ECG patterns for prognostication.

Nursing professionals can substantially contribute to patient care and outcomes in managing AMI patients with CS. They participate in the evaluation, observation, and management of patients' hemodynamic status, closely monitoring vital signs, cardiac rhythms, and therapeutic responses, administering medications like vasopressors and inotropic agents, and adjusting doses based on the patient's response. Moreover, nurses collaborate with the multidisciplinary team to ensure a seamless continuum of care, helping patients and their families understand their disease, treatment options, and lifestyle changes by providing education and emotional support ^13^.

This study aims to assess the differences in the characteristics and outcomes of STEMI and NSTEMI patients presenting with CS.

**METHODS**

**Research Question:**

What is the difference in characteristics and outcomes in adults (>18 years) diagnosed with STEMI and NSTEMI CS?

The latest Preferred Reporting Items for Systematic Reviews and Meta-Analyses (PRISMA) framework, published in 2020, was used for reporting ^14^. Ethical approval was not required since this review compiled the data that was available freely in selected databases.

**Literature search:**

The literature search was conducted in Medline, ScienceDirect, and Google Scholar databases. English-language articles that were freely available from inception till March 2025 were included. Prospective and retrospective studies (cohort, case-control, and cross-sectional studies) of cardiogenic shock patients, as well as studies reporting baseline characteristics and relevant outcome parameters, were included. Additionally, studies reporting data on STEMI and NSTEMI patients were included. Conference abstracts, case reports, narrative reviews, randomised control trials, and editorials were excluded. All relevant characteristics and clinical outcomes reported in the included studies were extracted.

CS in all included studies was defined based on the previously recommended standard definition ^15^. Patients with CS were further divided into two categories, STEMI-CS and NSTEMI-CS, based on ECG findings ^16^.

The latest universal definition of MI was used for the diagnosis of AMI ^17,18^. Any other electrocardiographic patterns in AMI patients were classified as NSTEMI ^18^.

**Collected data and outcomes of interest**

The review included all baseline characteristics that were reported in common from the included studies such as age, female gender, heart rate, body mass index (BMI), Acute Physiology and Chronic Health Evaluation (APACHE) score, Sequential Organ Failure Assessment (SOFA) score, Left ventricular ejection fraction (LVEF), troponin levels, comorbidities like current diabetes status, Charlton comorbidity index (CCI), prior myocardial infarction, and previous heart failure.

The primary outcomes considered for the study were in-hospital mortality and mortality in the coronary care unit intensive care unit (CCUICU). The secondary outcomes included incidence of sepsis, respiratory failure, cardiac arrest, heart failure, invasive and non-invasive ventilator use, PCI use, length of CICU, and hospital stay in days.

**Search strategy**

Databases were screened using the medical subject heading (MeSH) terms: “Cardiogenic shock” AND “Baseline characteristics” AND “Outcomes” OR “Clinical outcomes” AND “STEMI” OR “NSTEMI” AND “Observational studies” OR “Retrospective studies OR “Prospective studies” along with free text terms as a filter (**Supplementary File 1).**

**Selection of studies**

The primary investigators conducted the search, screened the titles and abstracts, and extracted the full text of relevant articles. The secondary investigators then extracted information on all baseline characteristics and clinical outcomes. The authors resolved any disagreement by discussion.

**Data extraction**

Two reviewers independently extracted study characteristics (authors, year, design, sample size, setting), patient demographics (age, sex, comorbidities), intervention details (STEMI‐CS vs. NSTEMI‐CS definitions), and outcomes (in‐hospital mortality, PCI use) using a pilot‐tested, standardized data‐collection form. Discrepancies were resolved by discussion between the two reviewers. All extraction decisions including resolution of ambiguous or inconsistent entries were logged in a study‐specific audit trail. For studies reporting incomplete or unclear outcome data, we first contacted corresponding authors via e‐mail (up to two attempts) to request missing values.

**Risk of bias assessment in included studies:**

The two independent investigators assessed the risk of bias using the Newcastle-Ottawa Scale (NOS) for observational studies. This scale comprises 7 items, classified into 3 domains: selection, comparability, and outcome, with the maximal score of 9 ^19^.

**Statistical analysis and bias assessment**

Microsoft Excel was used for data entry, and STATA 14.2 was used for analysis. Pooled differences were evaluated for continuous variables among STEMI and NSTEMI cases using the inverse variance method, which involved the standardized mean difference (SMD) and standard deviation. The differences in binary outcomes were summarized using Odds ratios (OR) with 95% confidence intervals (CI) were calculated by the Mantel-Haenszel method. Finally, the pooled estimate was reported as SMD or OR with 95% CI based on the variable type. The I^2^ statistic and the Chi-square test of heterogeneity were used to assess the between-study variance. The heterogeneity was categorized as mild (I^2^ < 25%), moderate (I^2^ 25-75%), and substantial when I^2^ >75%. To explore the source of heterogeneity, subgroup analysis was performed based on study region and meta-regression was performed based on potential covariates such as study region, study design, mean age, sample size, quality score and year of publication. A forest plot and Funnel plot were used to graphically represent the pooled prevalence and publication bias. Egger’s test was done to test the asymmetry of funnel plot and p-value less than 0.05 indicative of publication bias.

**RESULTS**

**Study selection**

Of 3133 identified articles, 1047 were removed as duplicates. An additional 1,751 articles were eliminated after the title and abstract screening. Of the remaining 335 studies, 324 were excluded after the full-text evaluation. One study was obtained from a citation search. Finally, 12 articles were incorporated in the study (20–31). Figure 1 explains the PRISMA 2020 flow diagram**.**

**Characteristics of the included studies**

Table 1 summarizes the key characteristics of the 12 included studies. The studies were conducted across a diverse range of geographical settings, including the USA, Taiwan, Spain, various European countries, Egypt, South Korea, and Germany, with sample sizes ranging from 239 to over 15,000 patients. Most of the studies (10 out of 12) were retrospective, while a few were prospective or post-hoc analyses of larger clinical trials or registries. The study settings varied, spanning single- and multicenter intensive care units, national databases, and specialized cardiac centers, with each study focusing on patients with AMI-CS in adults (typically ≥18 years).

Independent variables examined across studies included demographic profiles, clinical and laboratory parameters, hemodynamic and metabolic profiles, echocardiographic findings, and other relevant factors. Outcomes of interest were primarily centered on mortality metrics (in-hospital, 30-day, or long-term mortality), with several studies also reporting additional adverse events such as major bleeding, stroke, and rehospitalization rates.

As shown in Table 2, nine of the 12 included studies had high quality (NOS score ≥6).

**Difference in outcome parameters between STEMI-CS and NSTEMI-CS patients**

STEMI-CS and NSTEMI-CS patients did not vary much in the rates of in-hospital mortality (pooled OR 0.82, 95% CI: 0.52 to 1.30, (I^2^=98.7%, p <0.001) **[Figure 2]** need for PCI (pooled OR 1.97, 95% CI: 0.46 to 8.42, (I^2^=99.9%, p <0.001) **[Supplementary file 2]**, need for invasive ventilator (pooled OR 1.00, 95% CI: 0.70 to 1.45, (I^2^=84.6%, p<0.001) **[Supplementary file 3]**, and CICU stay duration in days (SMD of -0.22 (95% CI: -0.48 to 0.04, (I^2^=86.5%, p <0.001) **[Supplementary file 4]**. However, STEMI-CS patients had a significantly lower incidence of sepsis (pooled OR 0.40, 95% CI: 0.29 to 0.57, (I^2^=42.8%, p 0.19) **[Supplementary file 5]**, and required shorter hospital stay (SMD of -0.54 (95% CI: -0.77 to -0.31, I^2^=99.1%, p<0.001) **[Figure 3]** than NSTEMI-CS. There was a significant variability in the rates of cardiac arrest (pooled OR 1.78, 95% CI: 0.98 to 3.24, (I^2^=84.3%, p<0.001) **[Supplementary file 6]**.

**Difference in baseline characteristics between STEMI-CS and NSTEMI-CS patients**

STEMI-CS patients had a significantly lower age of presentation when compared to NSTEMI-CS cases, with the pooled SMD of -0.54 (95% CI: -0.67 to -0.42, (I^2^=96.9%, p <0.001) **[Figure 4].** Female gender distribution (pooled OR 0.96, 95% CI: 0.80 to 1.16, (I^2^=89.4%, p<0.001) **[Supplementary file 7]**, LVEF distribution (SMD of 0.02 (95% CI: -0.27 to 0.31, (I^2^=95.7%, p<0.001) **[Supplementary file 8]**, heart rate (SMD of -0.23 (95% CI: -0.66 to 0.19, (I^2^=98.2%, p <0.001) **[Supplementary file 9]** were comparable between the STEMI-CS and STEMI-CS. However, the STEMI-CS group reported lesser incidence of prior heart failure (pooled OR 0.27, 95% CI: 0.20 to 0.36, (I^2^=60.8%, p 0.03), **[Figure 5]** prior MI (pooled OR 0.42, 95% CI: 0.28 to 0.64, (I^2^=88%, p <0.001) **[Figure 6]**, diabetes (pooled OR 0.36, 95% CI: 0.25 to 0.52, (I^2^=37.9%, p 0.15) **[Supplementary file 10]**, and lower BMI (WMD of -0.44 (95% CI: -0.80 to -0.07, (I^2^=16.1%, p 0.30) **[Supplementary file 11]**, when compared to the NSTEMI-CS patients.

**Publication bias:**

Publication bias assessment was done for in-hospital mortality outcomes. The funnel plot (**Supplementary file 12**) showed symmetry among the included studies, as confirmed by Egger’s test (p = 0.31), indicating the absence of publication bias.

**Subgroup analysis and meta-regression**

***In-hospital mortality***

In subgroup analyses by WHO region, the pooled OR for in-hospital mortality in STEMI-CS versus NSTEMI-CS was 0.894 (95% CI 0.402–1.989; I² = 99.6%) in America, 0.815 (95% CI 0.574–1.157; I² = 37.8%) in Asia, and 0.699 (95% CI 0.277–1.768; I² = 91.3%) in Europe. The test for between-region differences was non-significant (Q = 0.16, df = 2, p = 0.925), indicating no evidence of effect modification by region (**Supplementary file 13**). Region did not significantly moderate mortality (Adj R² = –30.8%; joint p = 0.925), indicating no explained heterogeneity. Study design was non-significant (Adj R² = 4.4%; p = 0.248). Quality score was the only significant predictor (Adj R² = 44.5%; p = 0.045), explaining nearly half the residual variance. Mean age had no effect (Adj R² = –5.1%; p = 0.380), nor did sample size (Adj R² = –12.0%; p = 0.643). Year of publication together explained 46.1% of between-study variance but was not significantly associated with effect size (joint p = 0.209).

***Need for PCI***

In region-specific analyses of PCI use, the pooled OR for STEMI-CS versus NSTEMI-CS was 2.157 (95% CI 0.260–17.883; I² = 99.9%) in America, 1.665 (95% CI 0.728–3.811; I² = 74.5%) in Asia, and 1.784 (95% CI 0.173–18.441; I² = 94.2%) in Europe. The test for between-region differences was non-significant (Q = 0.05, df = 2, p = 0.975), indicating no evidence of effect modification by region (**Supplementary file 14**). Meta-regression did not show significant association with any variables or explain the heterogeneity based on any of the covariates (study region, sample size, mean age, year of publication, quality score and study design).

Subgroup analysis and meta-regression could not be performed for need of invasive ventilation and CICU duration due to limited number of studies.

***Sensitivity analysis***

Sensitivity analysis (**Supplementary file 15-18**) showed that there was no significant variation in the pooled effect size due to single study effects, indicating the studies are robust to outliers.

**DISCUSSION**

The study showed similar incidences of in-hospital mortality in STEMI-CS and NSTEMI-CS patients. However, STEMI-CS was associated with significantly lower rates of sepsis and cardiac arrest and shorter length of hospital stay. Additionally, there was a considerable difference in baseline characteristics such as age, prior history of heart failure and MI, BMI, and diabetes disease status between the STEMI-CS and NSTEMI-CS groups.

Studies showed that NSTEMI patients present with more cardiovascular risk factors ^10,20^, probably due to extensive coronary involvement. However, such patterns have not yet been reported in AMI-CS patients. Furthermore, this is the first review to explore different clinical characteristics and outcomes in STEMI and NSTEMI patients with complicating CS, utilizing the highest form of evidence.

In-hospital mortality was comparable in the two types of patients in this study. This result confirms the finding by Pahuja et al ^21^, which also reported comparable mortality rates across both study groups. However, it is important to note the significant heterogeneity observed across the studies (I2=89.0%), indicating substantial variability. The lack of consensus regarding the impact of MI type on mortality in CS patients warrants further investigation and may be attributed to variations in patient characteristics, treatment strategies, and follow-up protocols across different healthcare settings.

This study demonstrated that NSTEMI-CS patients presented at a younger age than STEMI-CS patients. This finding suggests that NSTEMI-CS primarily affects older individuals and is consistent with earlier studies ^4,22^. As advancing age is frequently associated with higher comorbidities and fragility, the greater age of NSTEMI-CS patients may influence treatment choices and resource allocation. Furthermore, this review demonstrated that, compared to the NSTEMI-CS group, STEMI-CS patients had a significantly lower incidence of prior heart failure, MI, diabetes, and a lower BMI. These findings suggest that STEMI-CS patients could have a relatively healthier baseline cardiovascular profile than NSTEMI cases. On the contrary, compared to STEMI-CS patients, NSTEMI-CS was linked to a higher incidence of sepsis and longer hospital admissions. This finding raises the possibility of a link between NSTEMI-CS and an increased systemic inflammatory response in the body, which could lead to worse outcomes and a longer recovery period.

The results indicate a low likelihood that selective reporting or small‐study effects materially influenced our summary estimates for secondary interventions. Nevertheless, we acknowledge that the relatively small number of studies available for certain endpoints may reduce the power of bias‐detection methods. Taken together, the absence of detectable publication bias across both primary and secondary outcomes strengthens the credibility and robustness of our meta‐analytic findings.

**Clinical implications**

Beyond statistical comparisons, these findings carry important clinical implications for the management of cardiogenic shock complicating STEMI versus NSTEMI. First, although the overall odds of in‐hospital mortality did not differ significantly between STEMI‐CS and NSTEMI‐CS (OR 0.82, 95% CI 0.52–1.30), the high absolute mortality rates observed in both groups (ranging from 20–50% in contemporary cohorts) underscore the need for rapid identification and escalation of care in all patients with myocardial infarction complicated by shock. Clinicians should therefore maintain a low threshold for early invasive monitoring (e.g., pulmonary artery catheterization) and initiation of vasopressor or inotropic support as soon as shock is recognized, regardless of infarct type.

Second, our subgroup analyses suggested considerable regional variability in PCI use—though differences were not statistically significant, practice patterns ranged from predominantly emergent angiography in Europe to more variable approaches in Asia and the Americas. From a clinical standpoint, this highlights the value of standardized “shock team” protocols, in which interventional cardiology, critical care, and cardiac surgery collaborate to ensure that mechanical circulatory support (e.g., intra‐aortic balloon pump, Impella, or extracorporeal membrane oxygenation) is deployed alongside revascularization when indicated. Such multidisciplinary models have been associated with improved survival in observational cohorts and are now incorporated into international guidelines for the management of STEMI complicated by shock.

Third, the strong inverse association between study quality and effect size in our meta‐regression (quality score β = –0.58, p = 0.045) serves as a reminder that real‐world clinical outcomes may be even more nuanced than those captured in retrospective registry analyses. High‐quality randomized data will be needed to clarify whether differential infarct‐related management strategies such as complete versus culprit‐only PCI, timing of revascularization, or use of advanced support devices translate into meaningful survival benefits. In the interim, clinicians should individualize revascularization decisions based on hemodynamic stability, infarct complexity, and comorbidity burden rather than relying solely on presentation subtype.

**Strengths and limitations:**

This review is the first to evaluate different baseline characteristics and outcomes of STEMI-CS and NSTEMI-CS. The review included 12 studies with a substantial sample size. Despite these strengths, the study has several limitations, including the high interstudy heterogeneity, which suggests potential variability in patient populations, interventions, and outcomes. Further studies across various healthcare settings should address the variability in treatment strategies, patient characteristics, and follow-up protocols.

**Conclusions and Recommendations:**

This study provides insight into the characteristics and outcomes of CS patients with different ECG parameters. While it reported similar in-hospital mortality in the two groups, substantial differences were observed in age, comorbidities, and clinical outcomes. These results further emphasize the importance of considering MI type while managing CS. Based on these findings, clinicians should carefully consider each patient's unique characteristics, such as age, past cardiovascular history, and comorbidities, when deciding the management of AMI-CS patients.

**Conflict of interest**

The authors report no conflicts of interest.

**Funding**

None

**Author’s Contribution**

ZS conceived and designed the study. ZS and BC collected the data and performed the

analysis. ZS was involved in the writing of the manuscript and is responsible for the integrity

of the study. Authors have read and approved the final manuscript.

**​****Data Availability Statement**

The datasets generated during and/or analyzed during the current study are available from the corresponding author on reasonable request.

**REFERENCES**

**1** Mechanic OJ, Gavin M, Grossman SA. Acute Myocardial Infarction. StatPearls. Treasure Island (FL): StatPearls Publishing, 2025. http://www.ncbi.nlm.nih.gov/books/NBK459269/ (accessed 3 Apr 2025).

**2** Reynolds HR, Hochman JS. Cardiogenic shock: current concepts and improving outcomes. *Circulation* 2008; **117**: 686–97.

**3** Berg DD, Bohula EA, van Diepen S, et al. Epidemiology of Shock in Contemporary Cardiac Intensive Care Units. *Circ Cardiovasc Qual Outcomes* 2019; **12**: e005618.

**4** Khalid L, Dhakam SH. A review of cardiogenic shock in acute myocardial infarction. *Curr Cardiol Rev* 2008; **4**: 34–40.

**5** Samsky MD, Morrow DA, Proudfoot AG, Hochman JS, Thiele H, Rao SV. Cardiogenic Shock After Acute Myocardial Infarction: A Review. *JAMA* 2021; **326**: 1840–50.

**6** Holmes DR, Berger PB, Hochman JS, et al. Cardiogenic shock in patients with acute ischemic syndromes with and without ST-segment elevation. *Circulation* 1999; **100**: 2067–73.

**7** Lechner I, Reindl M, Metzler B, Reinstadler SJ. Predictors of Long-Term Outcome in STEMI and NSTEMI-Insights from J-MINUET. *J Clin Med* 2020; **9**: 3166.

**8** Kim YH, Her A-Y, Rha S-W, et al. Comparison of Outcomes Between ST-Segment Elevation and Non-ST-Segment Elevation Myocardial Infarctions Based on Left Ventricular Ejection Fraction. *J Clin Med* 2024; **13**: 6744.

**9** Abbott JD, Ahmed HN, Vlachos HA, Selzer F, Williams DO. Comparison of outcome in patients with ST-elevation versus non-ST-elevation acute myocardial infarction treated with percutaneous coronary intervention (from the National Heart, Lung, and Blood Institute Dynamic Registry). *Am J Cardiol* 2007; **100**: 190–5.

**10** García-García C, Subirana I, Sala J, et al. Long-term prognosis of first myocardial infarction according to the electrocardiographic pattern (ST elevation myocardial infarction, non-ST elevation myocardial infarction and non-classified myocardial infarction) and revascularization procedures. *Am J Cardiol* 2011; **108**: 1061–7.

**11** Polonski L, Gasior M, Gierlotka M, et al. A comparison of ST elevation versus non-ST elevation myocardial infarction outcomes in a large registry database: are non-ST myocardial infarctions associated with worse long-term prognoses? *Int J Cardiol* 2011; **152**: 70–7.

**12** Goldberg RJ, Makam RCP, Yarzebski J, McManus DD, Lessard D, Gore JM. Decade-Long Trends (2001-2011) in the Incidence and Hospital Death Rates Associated with the In-Hospital Development of Cardiogenic Shock after Acute Myocardial Infarction. *Circ Cardiovasc Qual Outcomes* 2016; **9**: 117–25.

**13** McManus DD, Gore J, Yarzebski J, Spencer F, Lessard D, Goldberg RJ. Recent trends in the incidence, treatment, and outcomes of patients with STEMI and NSTEMI. *Am J Med* 2011; **124**: 40–7.

**14** Page MJ, McKenzie JE, Bossuyt PM, et al. The PRISMA 2020 statement: an updated guideline for reporting systematic reviews. *BMJ* 2021; **372**: n71.

**15** Hochman JS, Buller CE, Sleeper LA, et al. Cardiogenic shock complicating acute myocardial infarction--etiologies, management and outcome: a report from the SHOCK Trial Registry. SHould we emergently revascularize Occluded Coronaries for cardiogenic shocK? *J Am Coll Cardiol* 2000; **36**: 1063–70.

**16** McDonagh TA, Metra M, Adamo M, et al. 2021 ESC Guidelines for the diagnosis and treatment of acute and chronic heart failure. *Eur Heart J* 2021; **42**: 3599–726.

**17** Thygesen K, Alpert JS, White HD, Joint ESC/ACCF/AHA/WHF Task Force for the Redefinition of Myocardial Infarction. Universal definition of myocardial infarction. *Eur Heart J* 2007; **28**: 2525–38.

**18** Thygesen K, Alpert JS, Jaffe AS, et al. Fourth Universal Definition of Myocardial Infarction (2018). *J Am Coll Cardiol* 2018; **72**: 2231–64.

**19** Stang A. Critical evaluation of the Newcastle-Ottawa scale for the assessment of the quality of nonrandomized studies in meta-analyses. *Eur J Epidemiol* 2010; **25**: 603–5.

**20** Weiss ES, Chang DD, Joyce DL, Nwakanma LU, Yuh DD. Optimal timing of coronary artery bypass after acute myocardial infarction: a review of California discharge data. *J Thorac Cardiovasc Surg* 2008; **135**: 503–11, 511.e1-3.

**21** Pahuja M, Sinha SS, Kataria R, et al. Abstract 14086: Do Outcomes Differ Between STEMI and NSTEMI Cardiogenic Shock? *Circulation* 2022; **146**: A14086–A14086.

**22** Vallabhajosyula S, Prasad A, Gulati R, Barsness GW. Contemporary prevalence, trends, and outcomes of coronary chronic total occlusions in acute myocardial infarction with cardiogenic shock. *Int J Cardiol Heart Vasc* 2019; **24**: 100414.

**Figure captions**:

Figure 1: PRISMA 2020 flow diagram explaining the Search flow

Figure 2: Forest plot showing the difference in incidence of mortality between STEMI-CS and NSTEMI-CS

Figure 3: Forest plot showing the difference in hospital stay between STEMI-CS and NSTEMI-CS

Figure 4: Forest plot showing the difference in age of mortality between STEMI-CS and NSTEMI-CS

Figure 5: Forest plot showing the difference in prior heart failure between STEMI-CS and NSTEMI-CS

Figure 6: Forest plot showing the difference in prior MI between STEMI-CS and NSTEMI-CS

**Supplementary file legends:**

Supplementary file 1: Search strategy

Supplementary file 2: Forest plot showing the difference in PCI among STEMI-CS and NSTEMI-CS

Supplementary file 3: Forest plot showing the difference in invasive ventilation among STEMI-CS and NSTEMI-CS

Supplementary file 4: Forest plot showing the difference in CICU stay among STEMI-CS and NSTEMI-CS

Supplementary file 5: Forest plot showing the difference in Sepsis among STEMI-CS and NSTEMI-CS

Supplementary file 6: Forest plot showing the difference in cardiac arrest among STEMI-CS and NSTEMI-CS

Supplementary file 7: Forest plot showing the difference in gender distribution among STEMI-CS and NSTEMI-CS

Supplementary file 8: Forest plot showing the difference in LVEF among STEMI-CS and NSTEMI-CS

Supplementary file 9: Forest plot showing the difference in heart rate stay among STEMI-CS and NSTEMI-CS

Supplementary file 10: Forest plot showing the difference in DM prevalence stay among STEMI-CS and NSTEMI-CS

Supplementary file 11: Forest plot showing the difference in BMI among STEMI-CS and NSTEMI-CS

Supplementary file 12: Funnel plot for publication bias assessment

Supplementary File 13: Subgroup analysis of in-hospital mortality by study region

Supplementary File 14: Subgroup analysis of need for PCI by study region

Supplementary File 15: Sensitivity analysis plot for in-hospital mortality

Supplementary File 16: Sensitivity analysis plot for need for PCI

Supplementary File 17: Sensitivity analysis plot for need for invasive ventilation

Supplementary File 18: Sensitivity analysis plot for CICU duration
