# Supplementary material for: Differences in the characteristics and outcomes of STEMI versus NSTEMI cardiogenic shock: A systematic review and meta-analysis
Source: Medicine (Baltimore). 2025 Oct 17;104(42):e44951. doi: 10.1097/MD.0000000000044951 (PMC12537256; doi:10.1097/MD.0000000000044951)

Supplementary file 2: Forest plot showing the difference in PCI among STEMI-CS and NSTEMI-CS


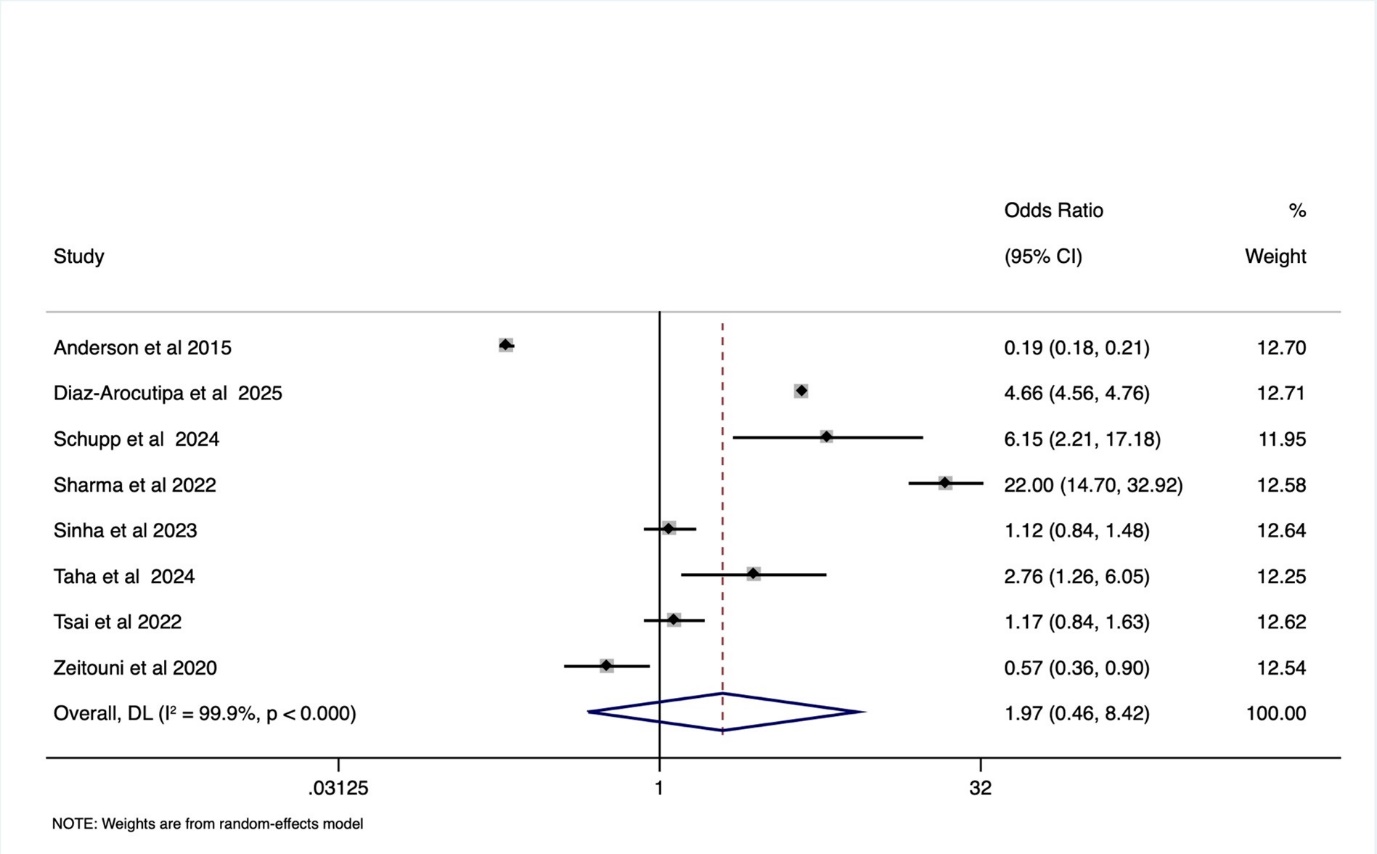


Supplementary file 3: Forest plot showing the difference in invasive ventilation among STEMI-CS and NSTEMI-CS


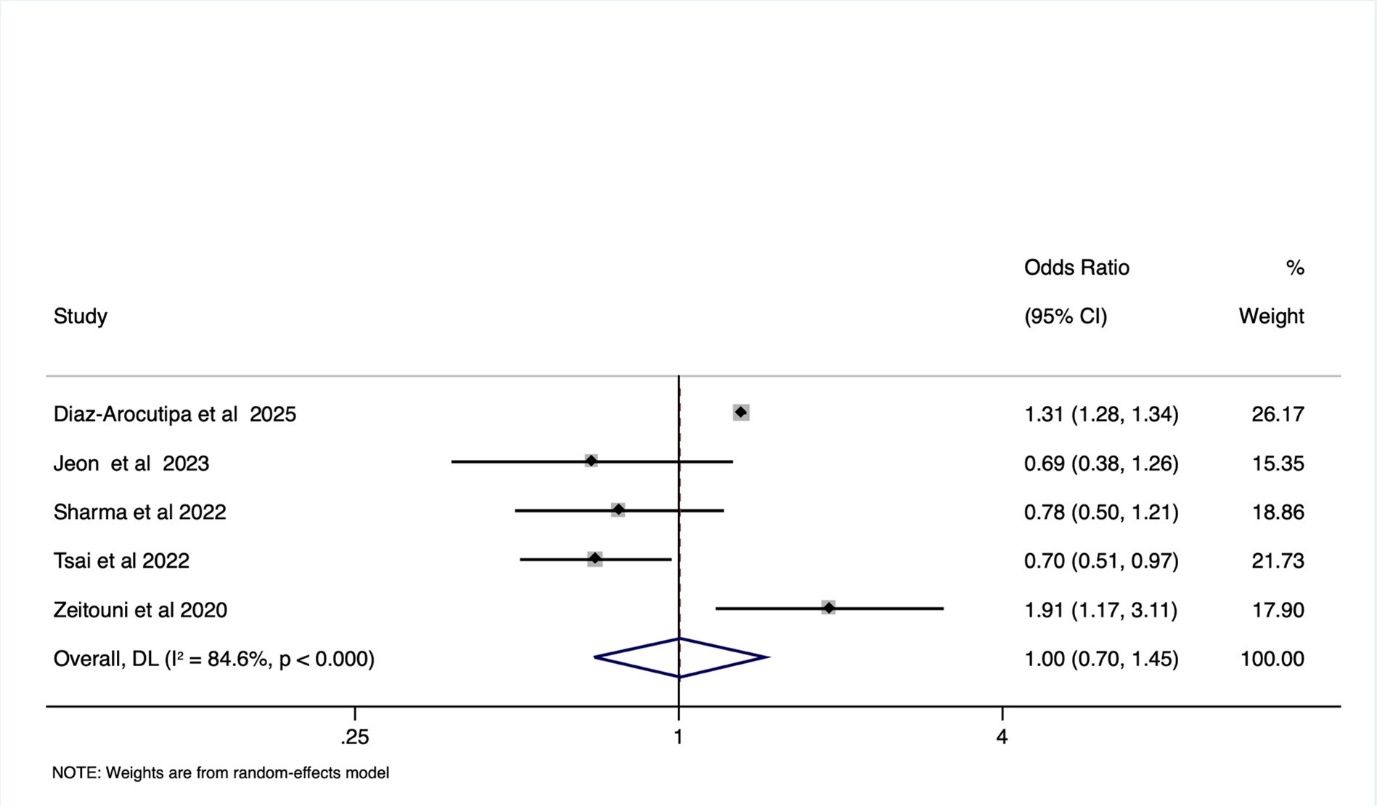


Supplementary file 4: Forest plot showing the difference in CICU stay among STEMI-CS and NSTEMI-CS


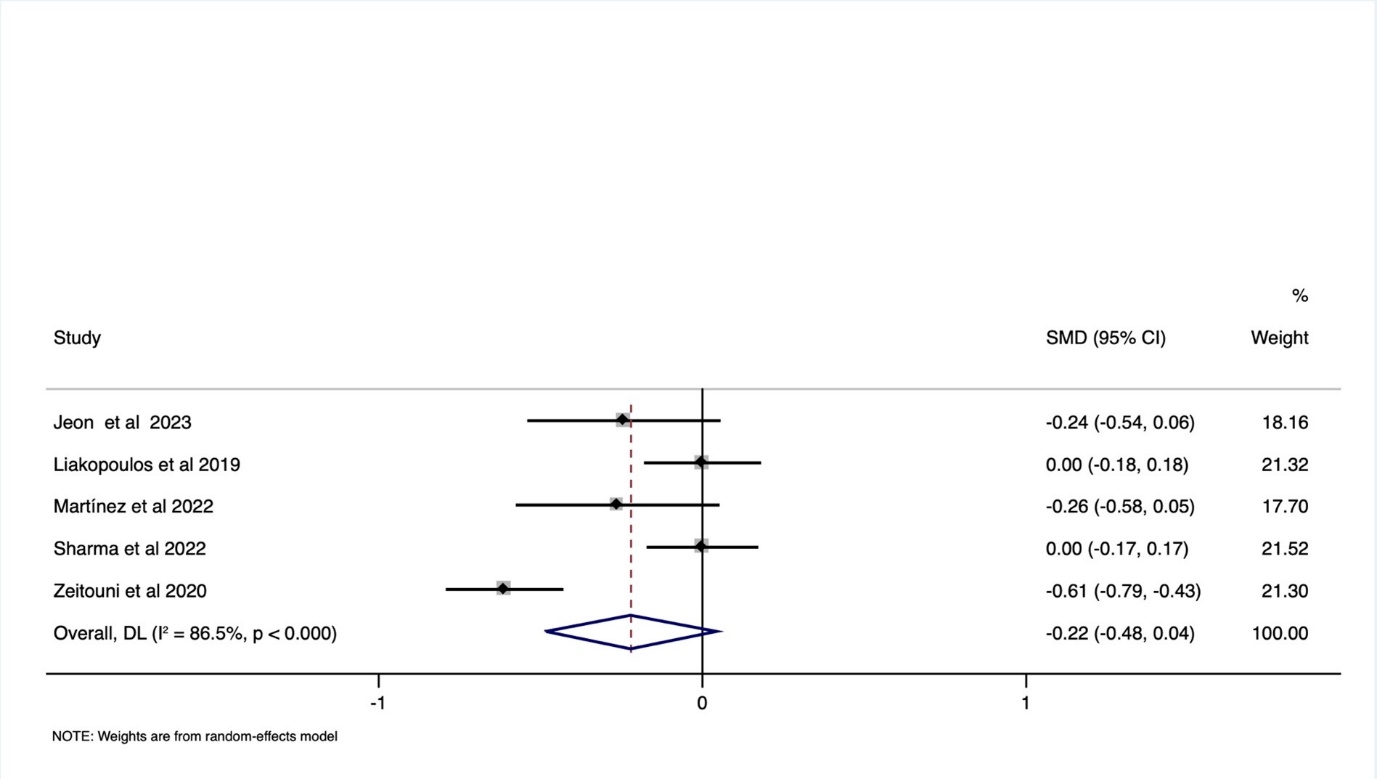


Supplementary file 5: Forest plot showing the difference in Sepsis among STEMI-CS and NSTEMI-CS


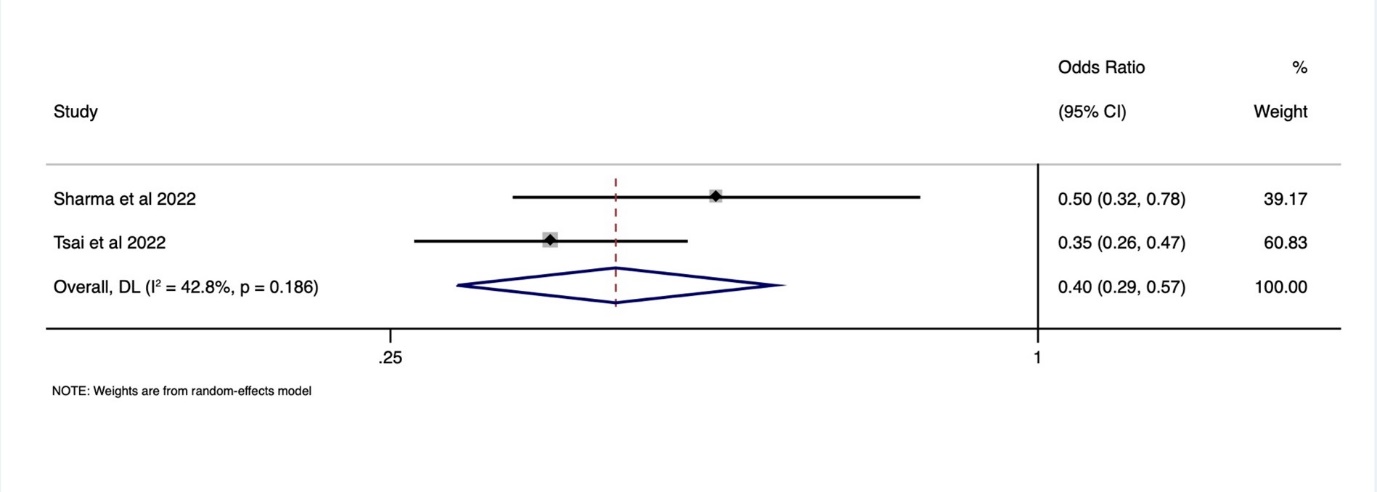


Supplementary file 6: Forest plot showing the difference in cardiac arrest among STEMI-CS and NSTEMI-CS


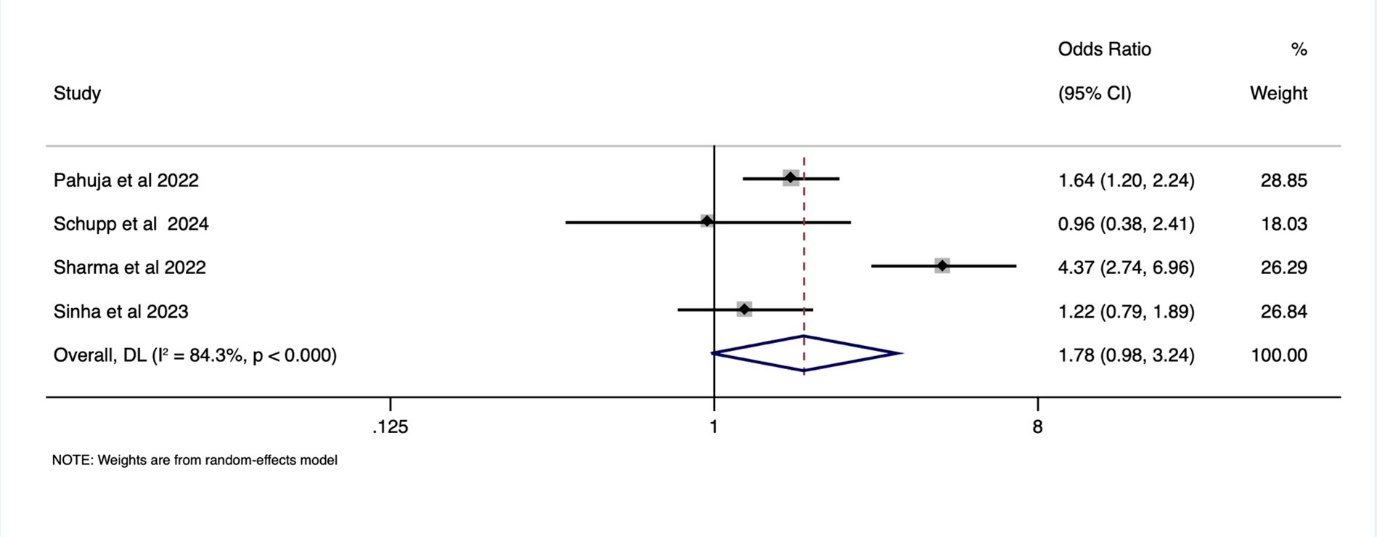


Supplementary file 7: Forest plot showing the difference in gender distribution among STEMI-CS and NSTEMI-CS


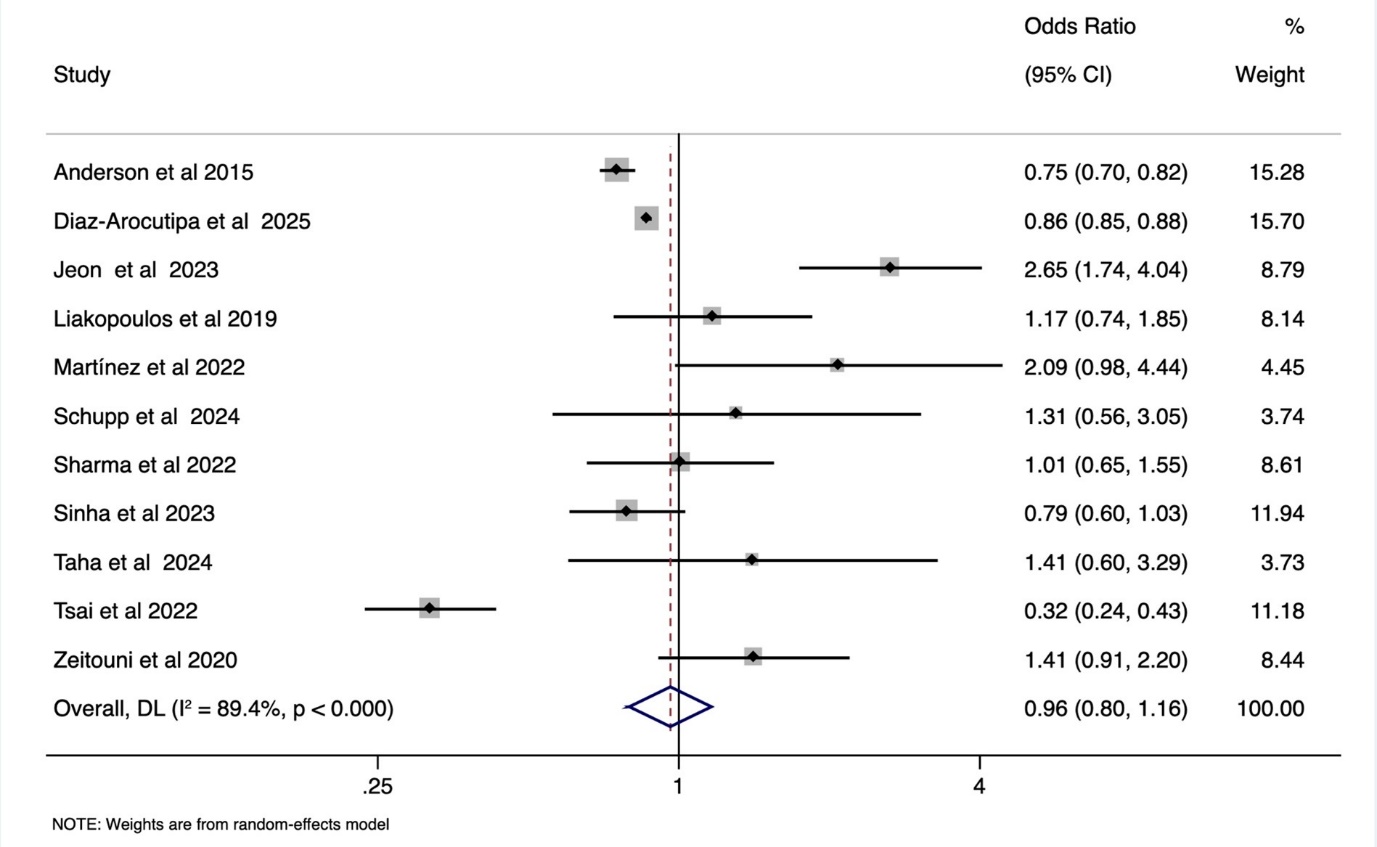


Supplementary file 8: Forest plot showing the difference in LVEF among STEMI-CS and NSTEMI-CS


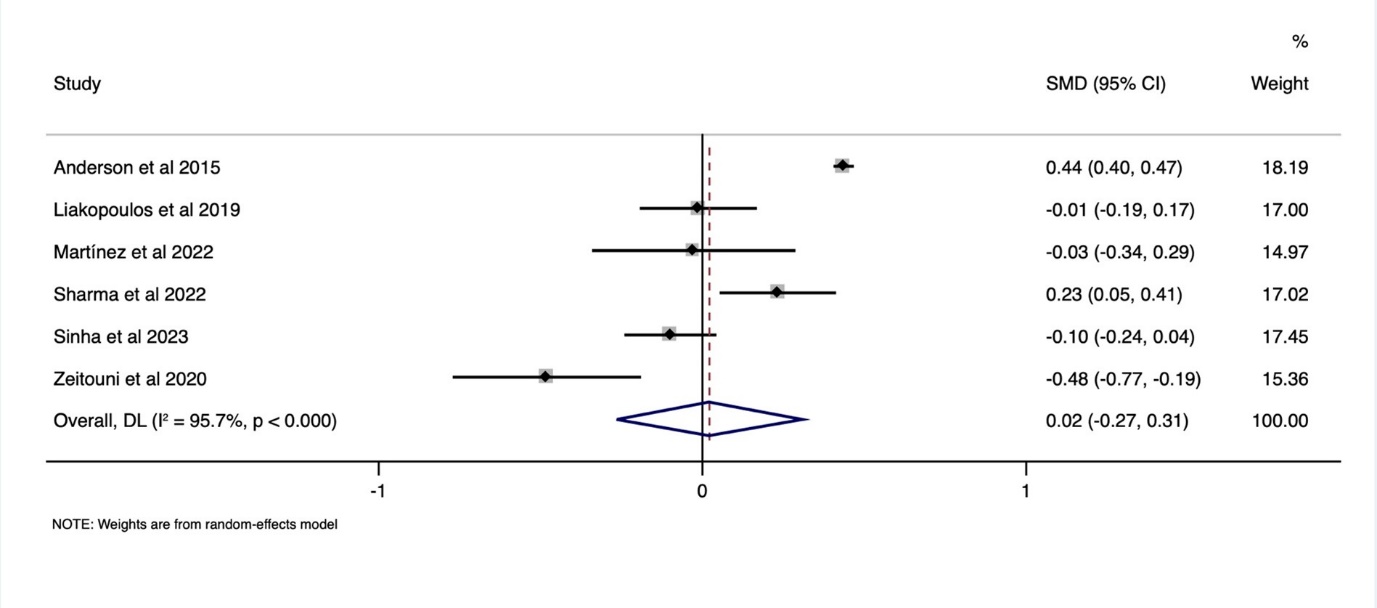


Supplementary file 9: Forest plot showing the difference in heart rate stay among STEMI-CS and NSTEMI-CS


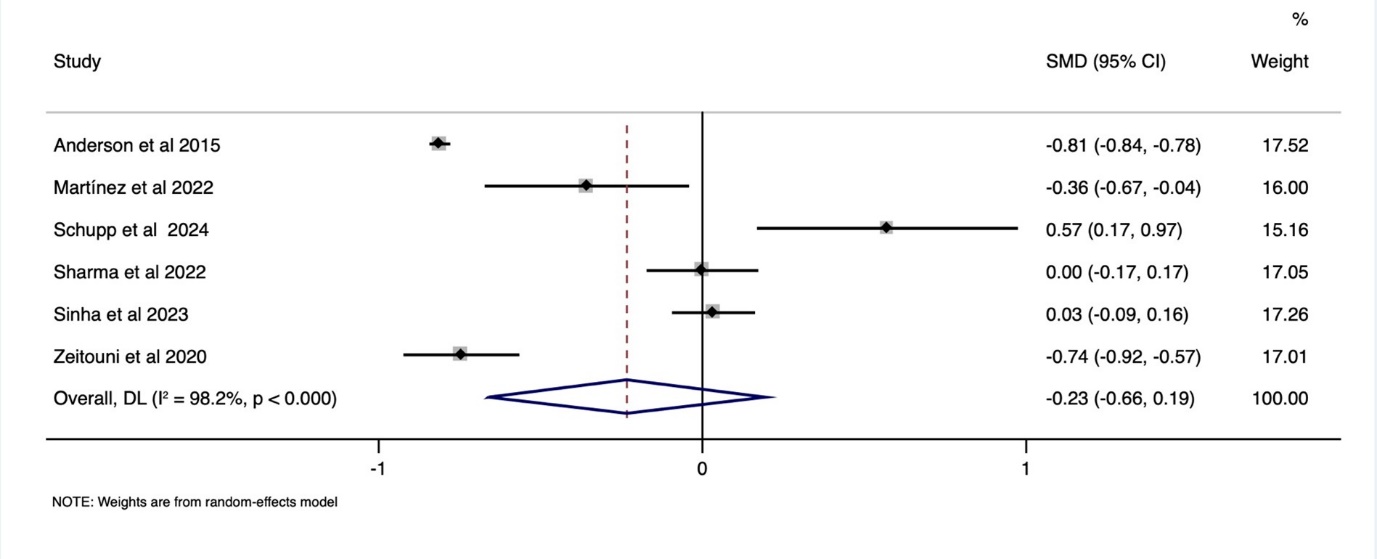


Supplementary file 10: Forest plot showing the difference in DM prevalence stay among STEMI-CS and NSTEMI-CS


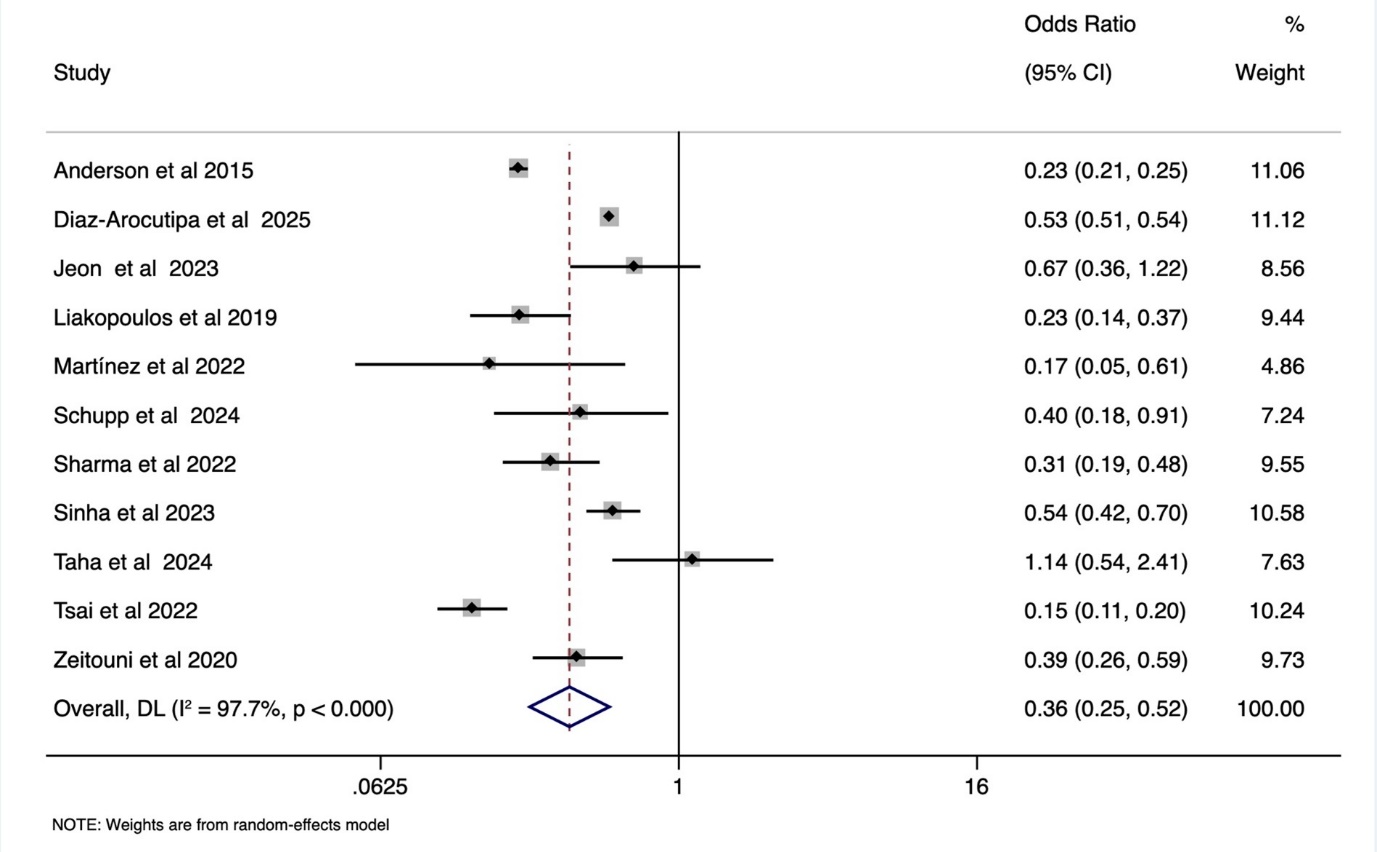


Supplementary file 11: Forest plot showing the difference in BMI among STEMI-CS and NSTEMI-CS


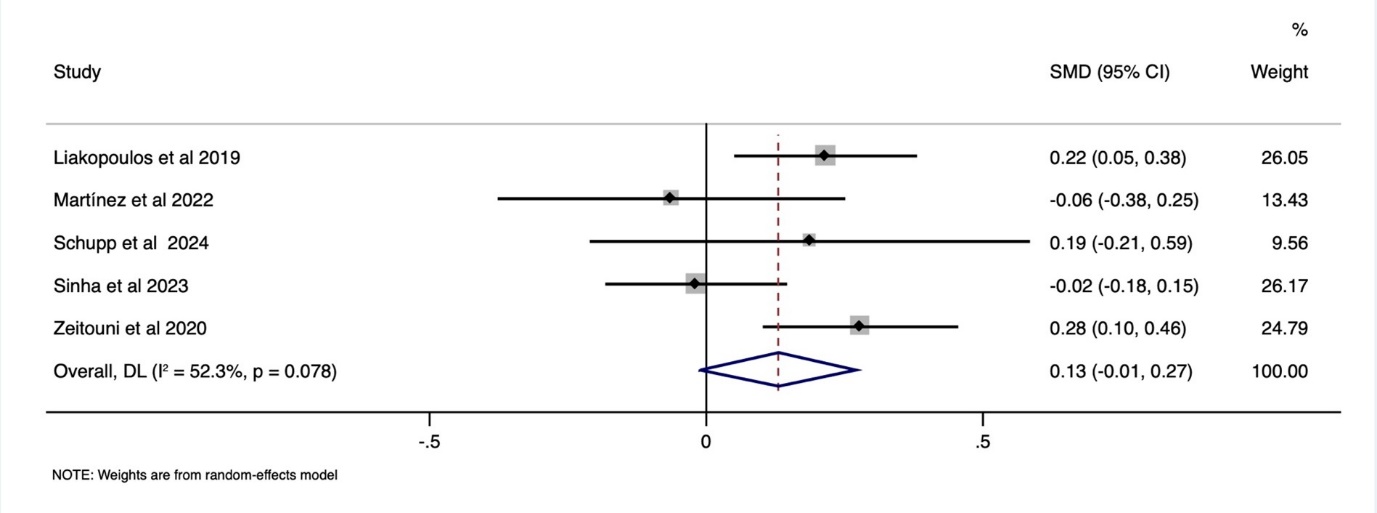


Supplementary file 12: Funnel plot for publication bias assessment


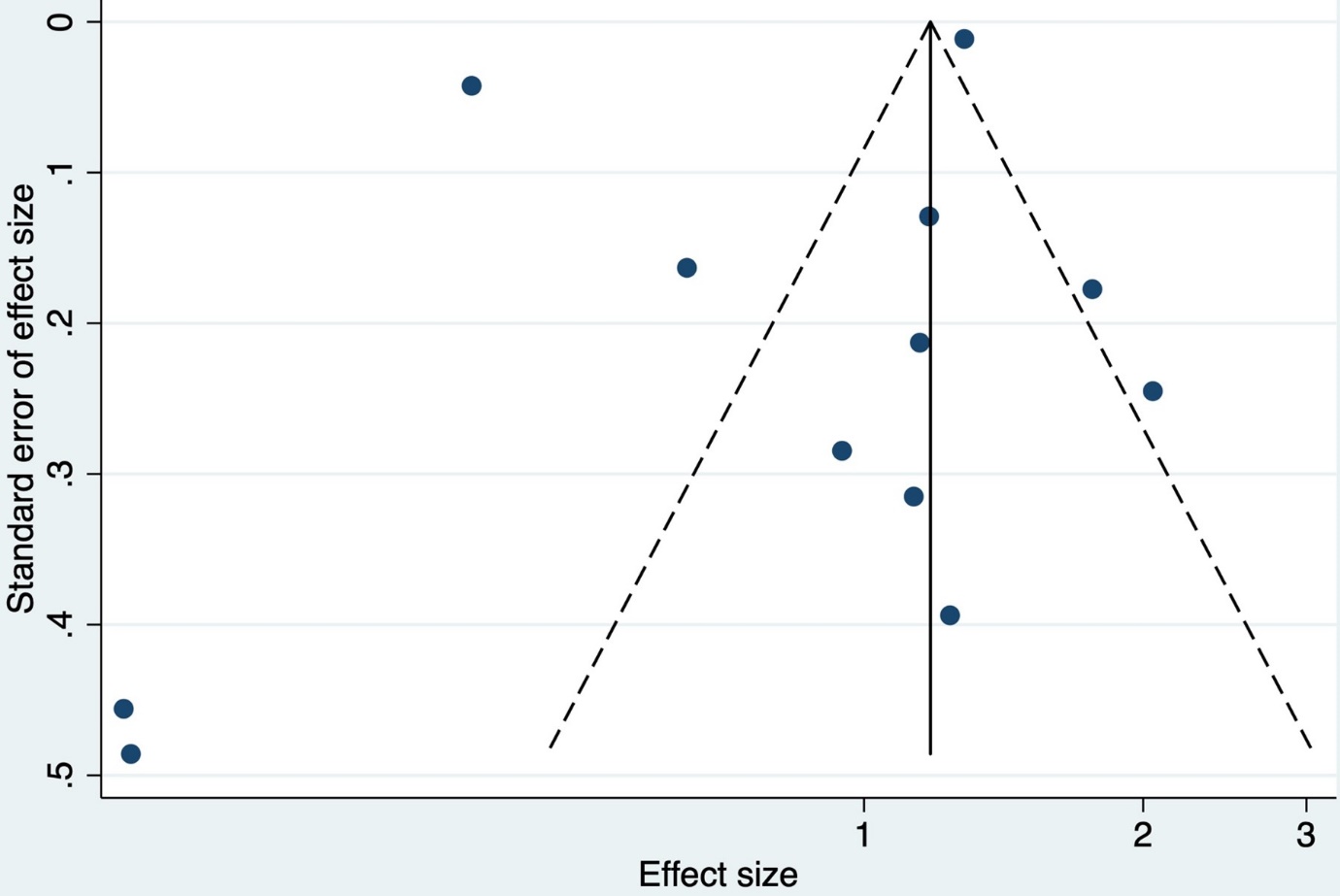


Supplementary File 13: Subgroup analysis of in-hospital mortality by study region


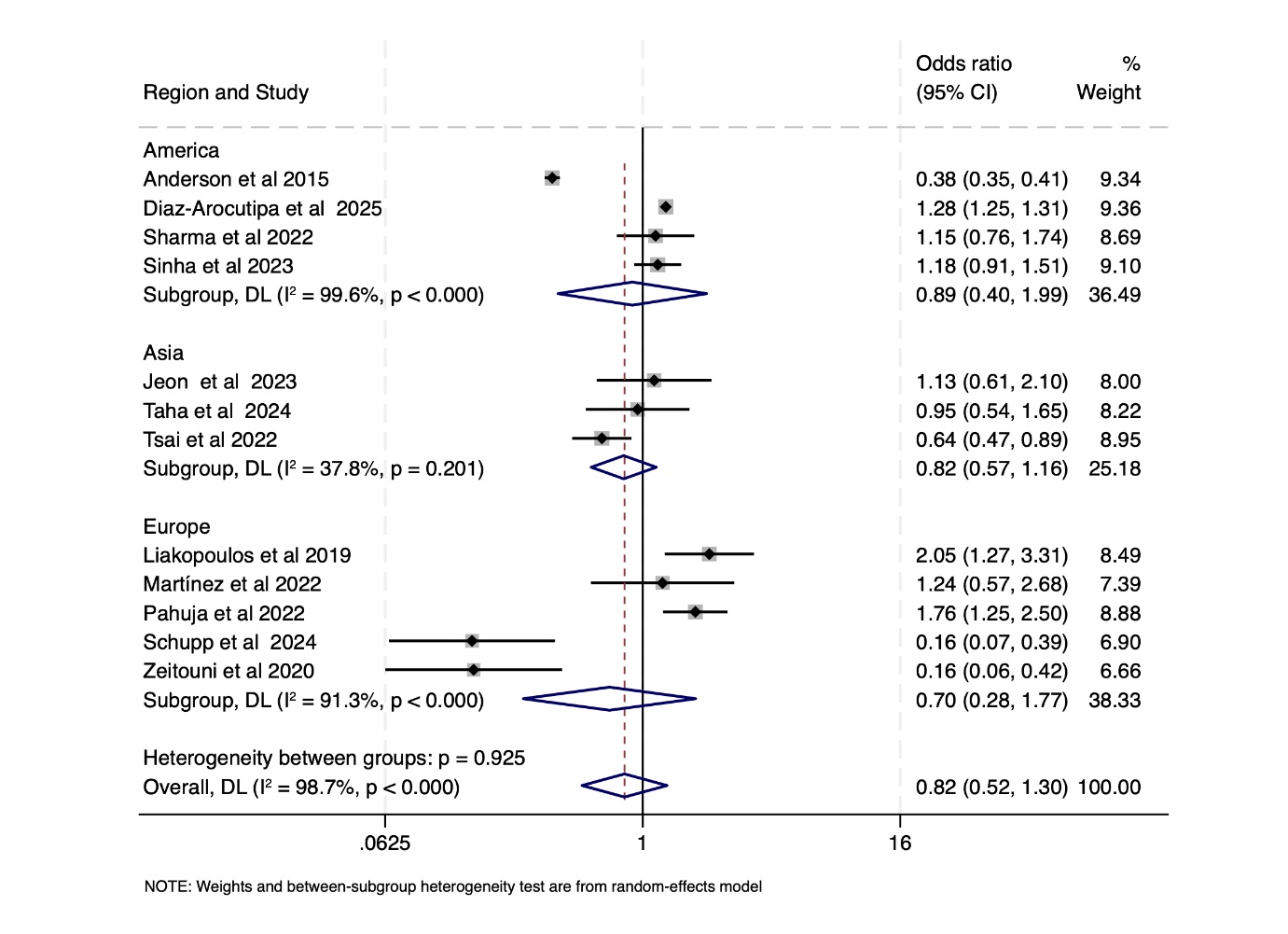


Supplementary File 14: Subgroup analysis of need for PCI by study region


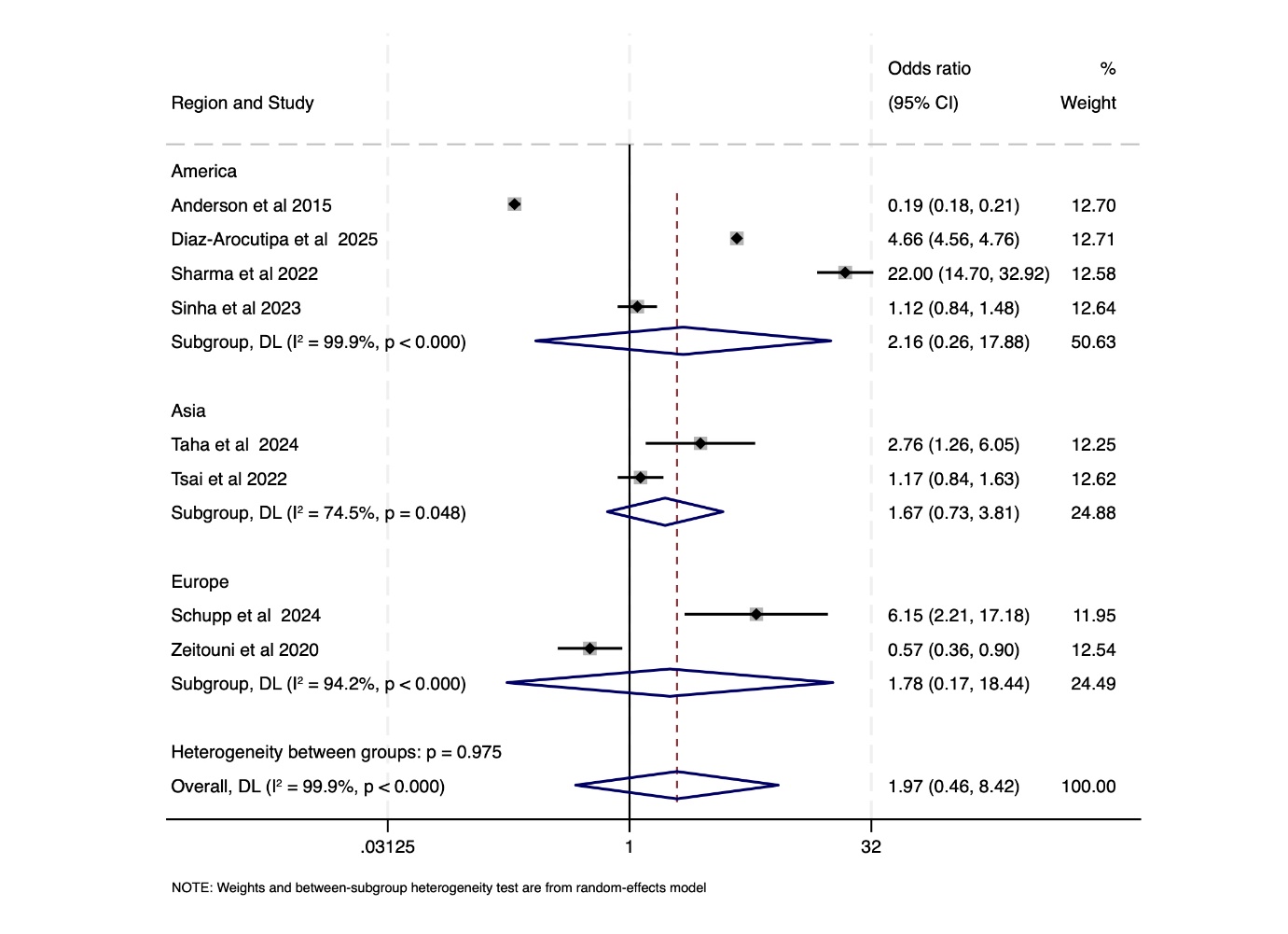


Supplementary File 15: Sensitivity analysis plot for in-hospital mortality


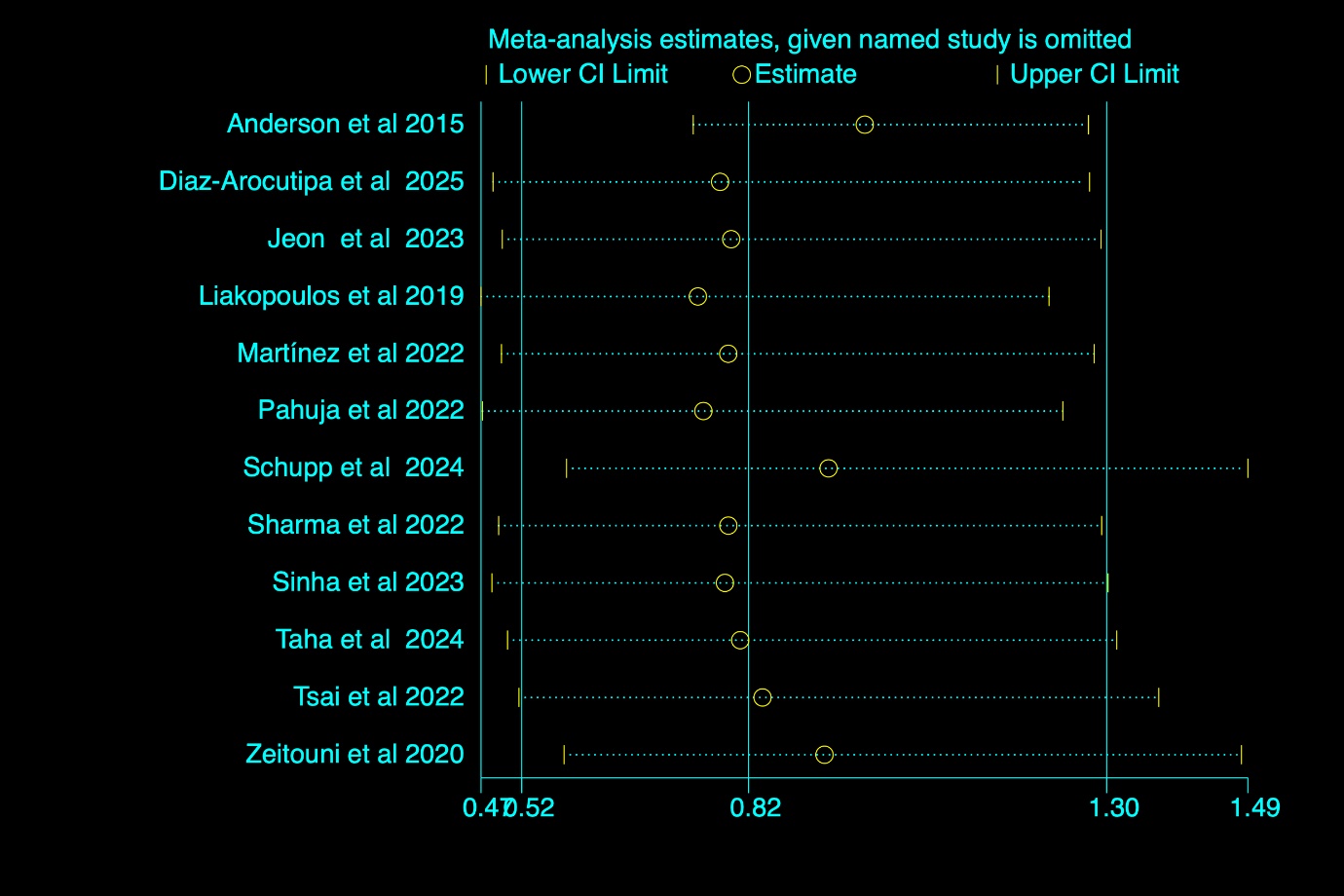


Supplementary File 16: Sensitivity analysis plot for need for PCI


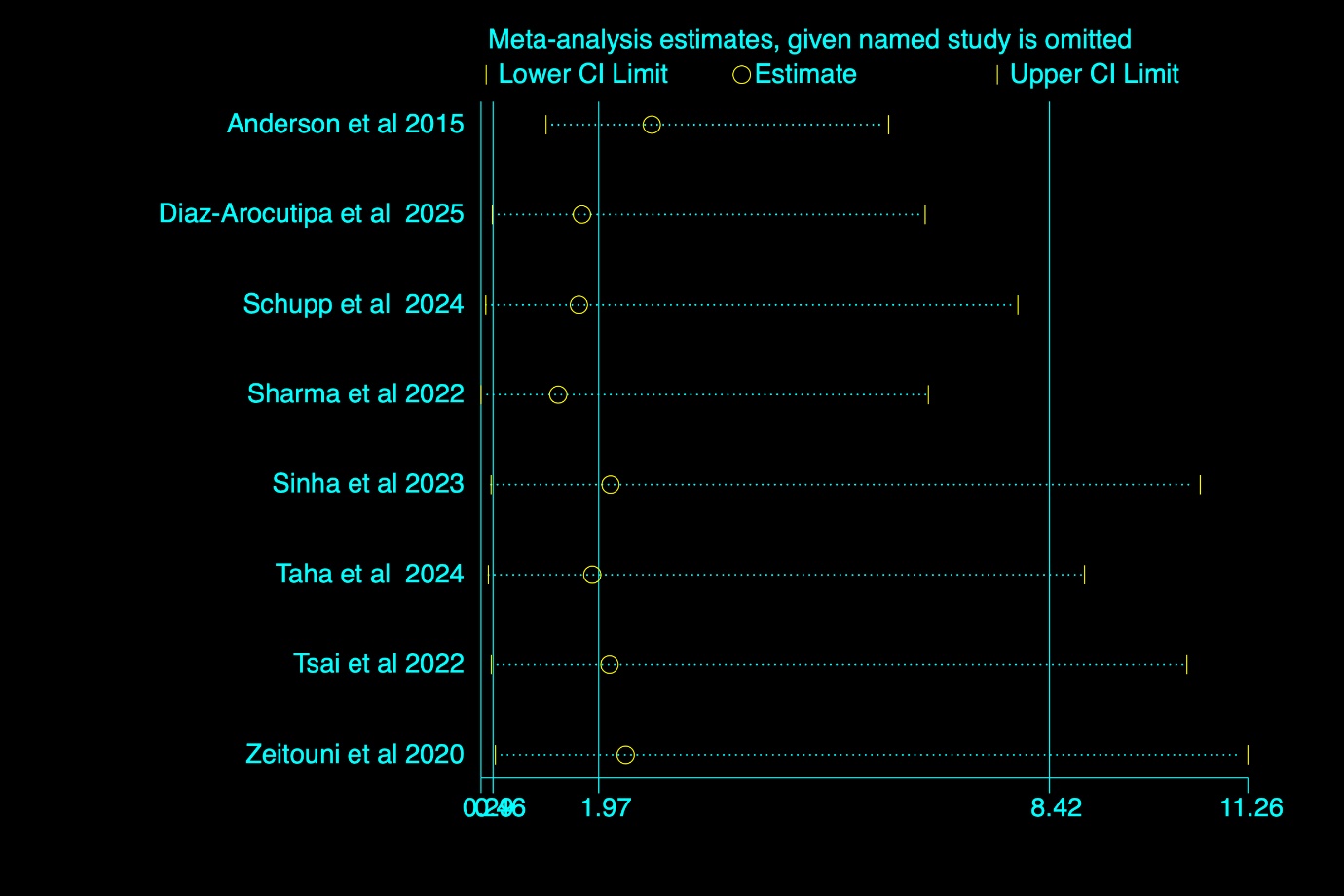


Supplementary File 17: Sensitivity analysis plot for need for invasive ventilation


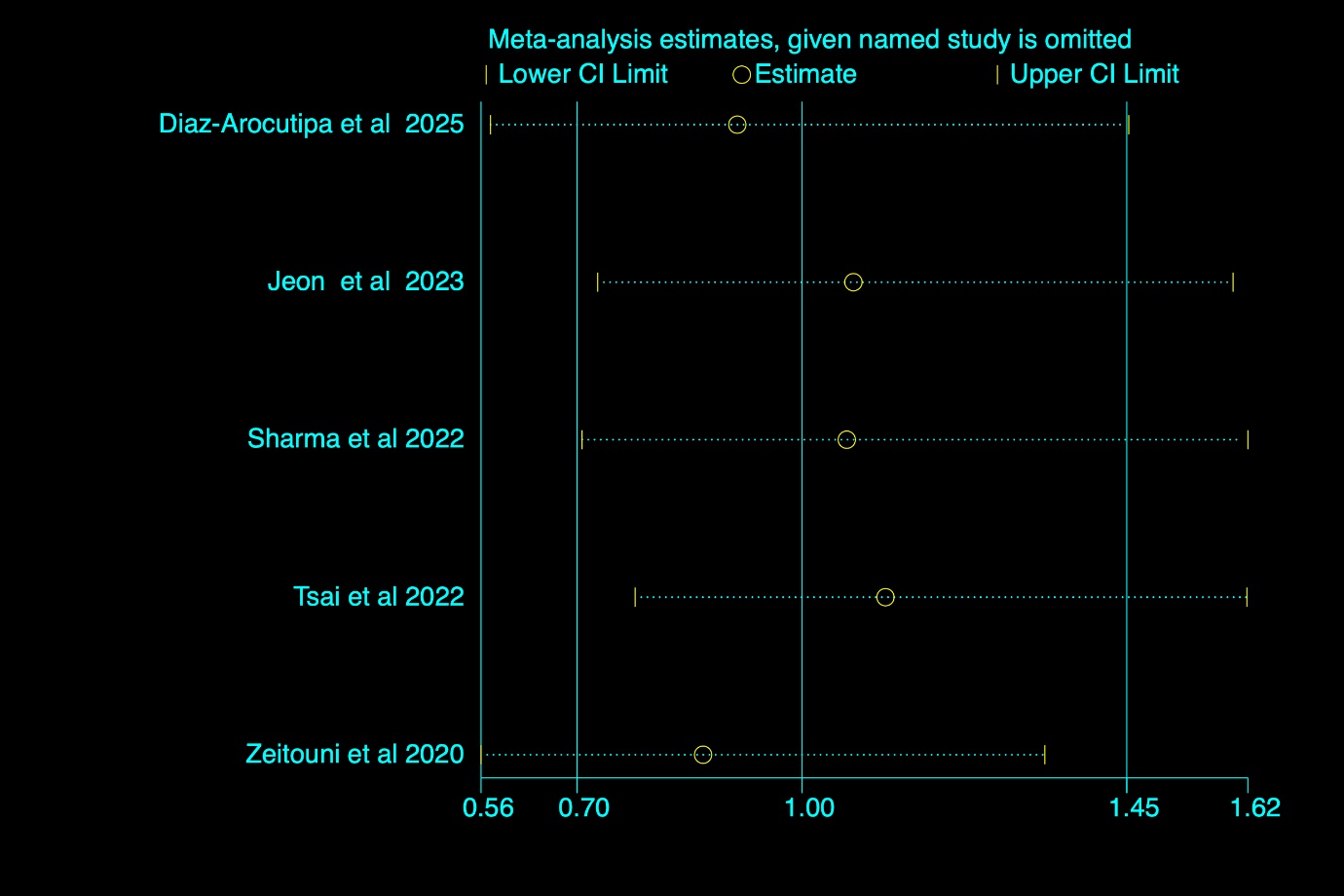


Supplementary File 18: Sensitivity analysis plot for CICU duration


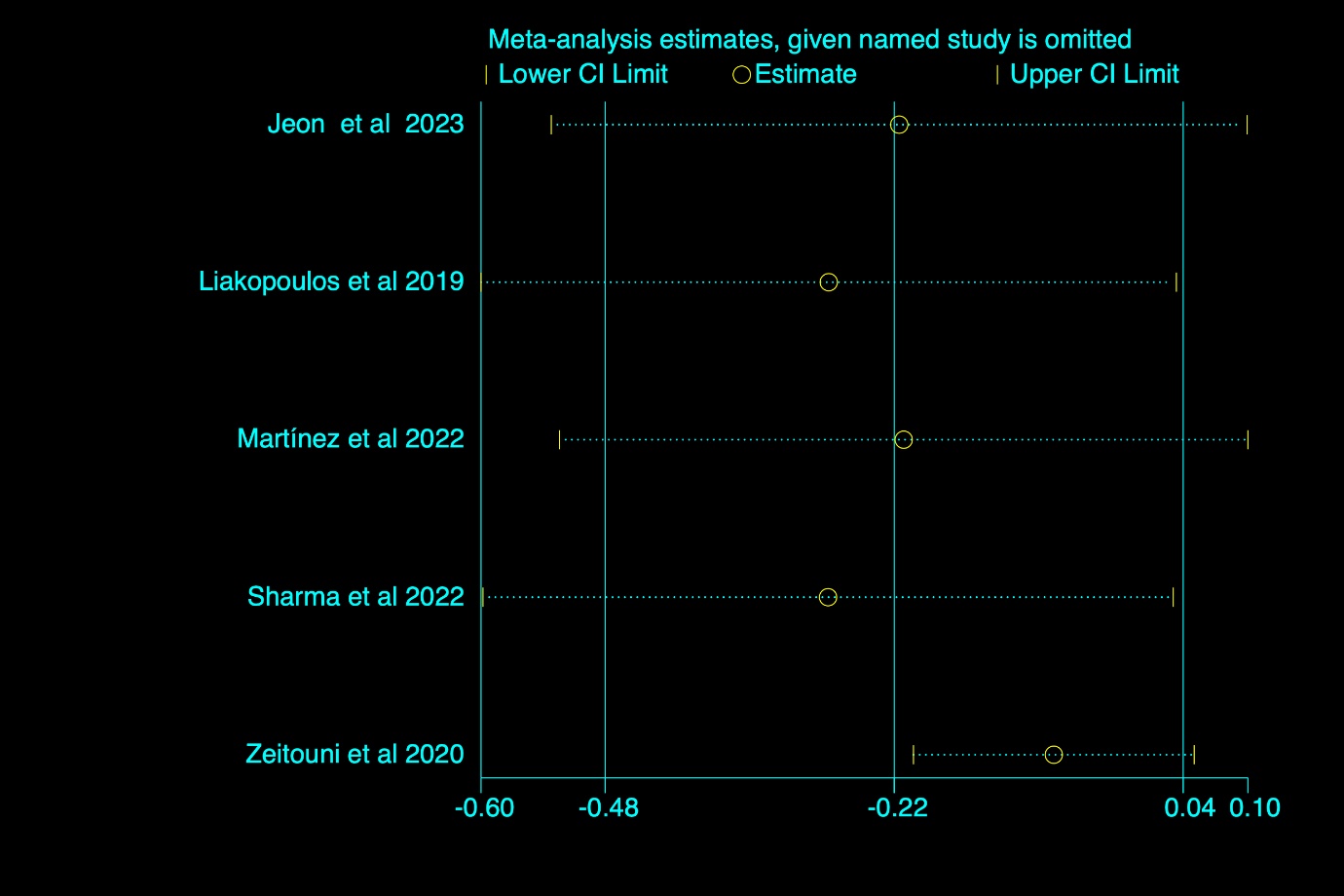

Supplement: Supplementary file 2 [file medi-104-e44951-s002.docx]
